# Total Synthesis of (+)-Linoxepin by Utilizing the Catellani Reaction**

**DOI:** 10.1002/anie.201302327

**Published:** 2013-04-16

**Authors:** Harald Weinstabl, Marcel Suhartono, Zafar Qureshi, Mark Lautens

**Keywords:** C–H functionalization, Catellani reaction, domino reactions, lignans, total synthesis

Lignans are a diverse class of plant-derived natural products belonging to the phytooestrogen family. They have long been used as herbal remedies for pain, rheumatoid arthritis, and warts.^1^ However, more recently, lignans exhibiting immunosuppressive activity, tumor growth inhibition, and anti-fungal properties have been used in disease therapy, such as the anticancer agent etoposide.^2^

In 2007, Schmidt and co-workers isolated a lignan from the aerial parts of *Linum perenne L.* (Linaceae) with a previously undescribed carbon skeleton, which they named linoxepin (**1**).^3^ This caffeic acid dimer exhibits an oxidation-prone dihydronaphthalene core, a tetrasubstituted double bond embedded within a highly strained ring system, and a dibenzo–dihydrooxepine moiety, which is unique within this class of molecules. These interesting structural characteristics and their associated challenges make (+)-linoxepin (**1**) an interesting synthetic target.

As means to address the tetrasubstituted aromatic A-ring embedded in the structure of **1**, we envisioned using the palladium-catalyzed Catellani reaction. This process would allow the installation of all of the carbon atoms needed to prepare compound **1**. The selective formation of multiple bonds in a single step has become an attractive way to construct highly complex frameworks that would be difficult to access using classical methods.^4^ Therefore, the use of transition-metal-catalyzed domino reactions is an interesting strategy for the assembly of complex natural products.

During the preparation of this manuscript, Tietze and co-workers published a ten-step synthesis of (±)-linoxepin (**1**) using an elegant palladium-catalyzed domino reaction to construct two of the five rings found in the natural product.^5^ Herein, we report the first asymmetric synthesis of (+)-**1** in eight steps using our modified version of the Catellani reaction.^6^

In this transformation, norbornene is used to facilitate an *ortho* C-H functionalization between an aryl iodide and an alkyl halide (Scheme 1). Along with methodology studies, our group has recently reported the use of this powerful reaction for the synthesis of molecular motors and highly substituted phenanthridines.^11^

**Scheme 1:**
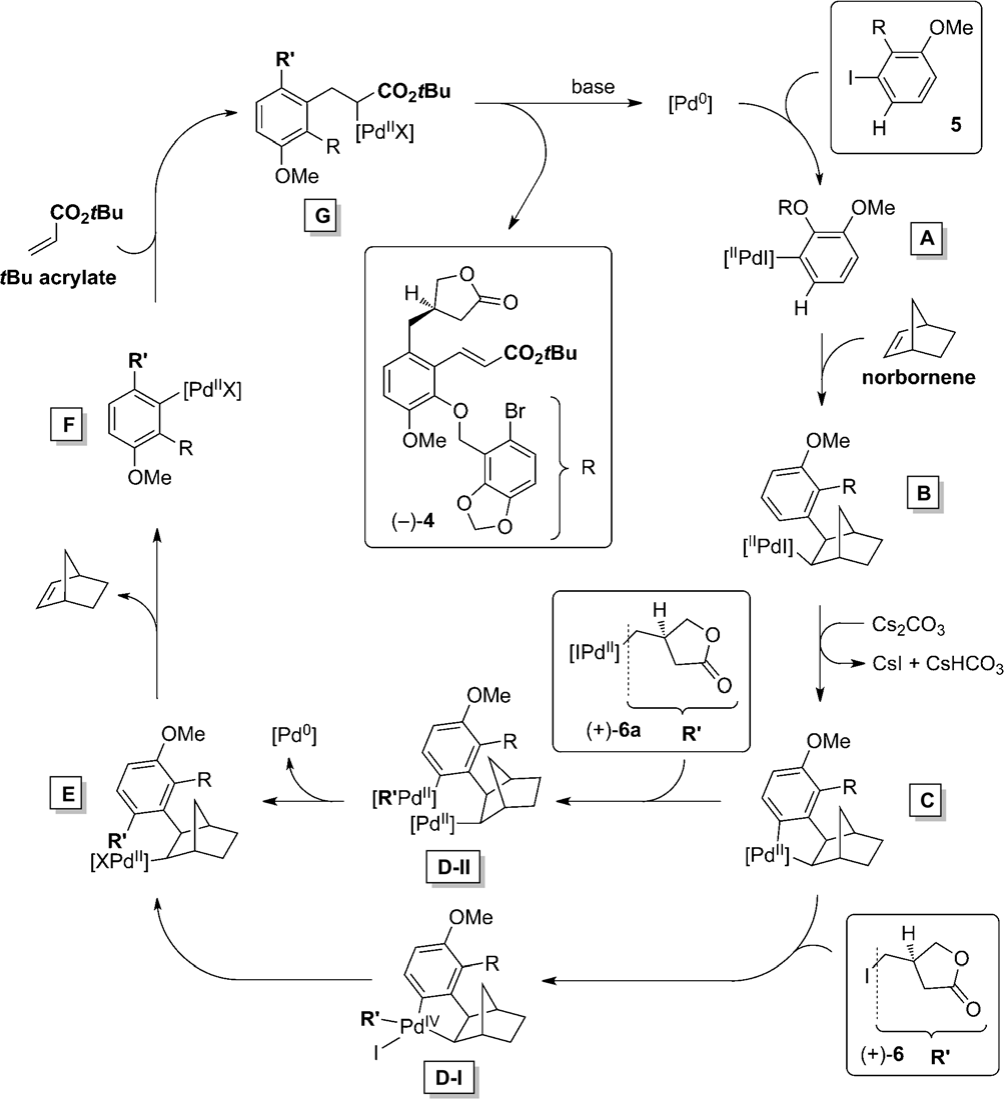
Proposed mechanism of the Catellani reaction.

The precise mechanism of this reaction is still under investigation, but it is known that the complex catalytic cycle is initiated by a Pd^0^ catalyst that oxidatively adds into the Ar–I bond of **5** to form intermediate (**A**), which then carbopalladates norbornene to furnish intermediate (**B**). The lack of a suitable β-hydrogen prevents *syn*-β-hydride elimination. Instead, base-mediated C–H activation occurs to form palladacycle (**C**), which may follow one of two pathways: Oxidative addition to alkyl halide **6** can produce Pd^IV^ intermediate **D-I**, which upon reductive elimination forms intermediate **E**.^9^ Another plausible sequence is a transmetalation between two different Pd^II^ centers (**D-II**), which forms **E** following reductive elimination.^10^ Expulsion of norbornene by retro carbopalladation affords intermediate (**F**). Finally, a Mizoroki–Heck type reaction completes the catalytic cycle, thus affording the desired caffeic acid derivative **4** in a single step.^7^–^10^

Our retrosynthetic analysis (Scheme 2) begins with the opening of the E-ring (**2**) followed by subsequent opening of the B-ring (**3**). Aldehyde **3** can be obtained from cinnamic acid derivative **4**, which would be obtained by the key Catellani reaction. Ether **5** could be obtained by the condensation of iodo-guaiacol **11** and benzyl iodide **10**. Iodolactone **6** is a known compound and can be synthesized in enantiomerically pure or racemic form by the procedure published by Zutter et al.^12^, ^13^

**Scheme 2:**
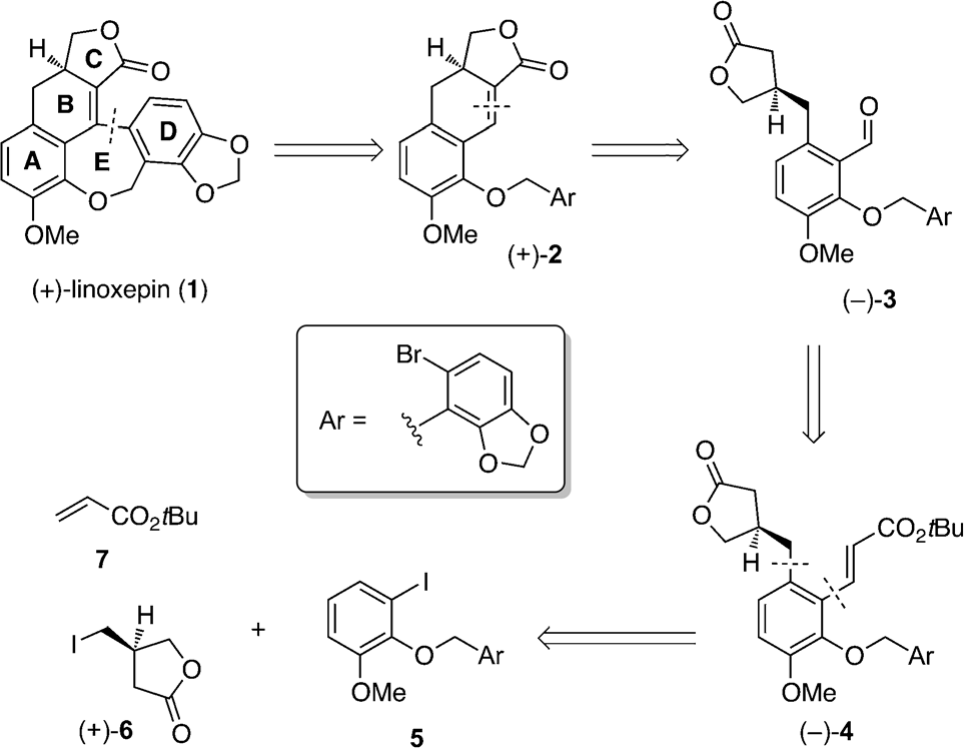
Retrosynthetic analysis of (+)-linoxepin (**1**).

Concurrent with our enantioselective synthesis, we undertook a racemic synthesis.^14^ Our synthesis began with the formylation of commercially available 5-bromobenzo[*d*][1,3]dioxole (**8**).^15^ The crude material was reduced with NaBH_4_ to the corresponding benzyl alcohol **9**. Treating **9** with TMSCl and NaI gave benzyl iodide **10** in quantitative yield. Directed *ortho* lithiation of guaiacol, followed by quenching with iodine, furnished intermediate **11**. Williamson ether synthesis delivered Catellani precursor **5** (Scheme 3).

**Scheme 3:**
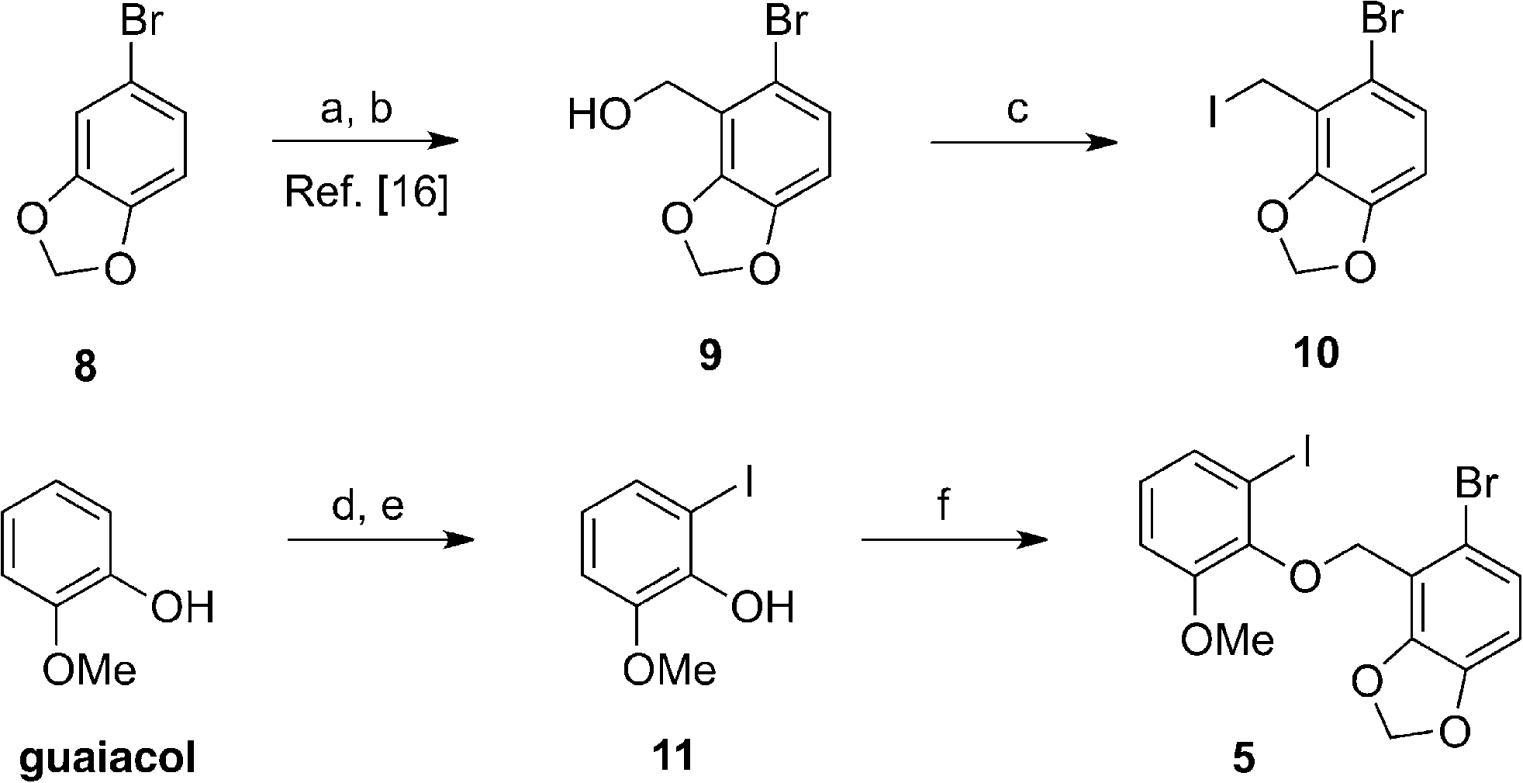
Synthesis of the aryliodide **5**. a) i) HN*i*Pr_2_, *n*BuLi, THF, −78 °C, 1 h; ii) DMF, THF, −78 °C→RT, 95 %; b) NaBH_4_, THF, reflux, 90 min, 97 %; c) TMSCl, NaI, CH_3_CN, RT, 1 h, 99 %; d) DHP, PPTS, CH_2_Cl_2_, RT, 4 h, 95 %; e) i) *n*BuLi, THF, RT, 7 h; ii) I_2_, THF, −50 °C→RT, 16 h, 98 %; f) **10**, K_2_CO_3_, acetone, reflux, 94 %. DHP=3,4-dihydro-2*H*-pyran; PPTS=pyridinium *p*-toluenesulfonate.

With aryl iodide **5** in hand, the stage was set for our key step. Under the previously optimized conditions, the formation of the desired tricycle (±)-**4** proceeded smoothly in 92 % yield of isolated product to furnish 860 mg of the advanced intermediate (Scheme 4).

**Scheme 4:**
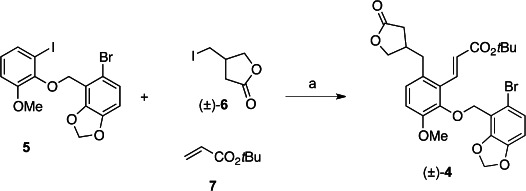
Key step: Catellani reaction. a) Pd(OAc)_2_ (10 mol %), PPh_3_ (22 mol %), norbornene, Cs_2_CO_3_, DMF (sealed tube), 90 °C, 5 h, 92 %.

Preliminary studies indicated that the formation of the tetra-substituted double bond was challenging,^16^ and so an aldol condensation was envisaged in the last step. When (±)-**4** was subjected to the Mizoroki–Heck conditions, oxepane **12** was formed in high yield (Scheme 5). However, all attempts at oxidative cleavage of the trisubstituted olefin failed and led to the formation of a complex mixture of products, which is presumably due to steric hindrance or the electron-rich aromatic ring. X-ray crystallographic analysis confirmed the extreme crowding in **12**.

**Scheme 5:**
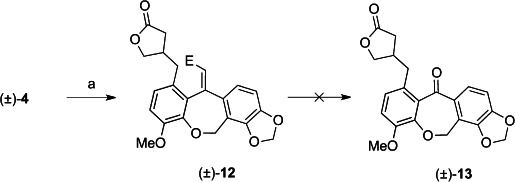
Attempted synthesis of (±)-linoxepin (**1**). a) PdCl_2_ (20 mol %), PPh_3_ (44 mol %) NEt_3_ (10 equiv), microwave radiation, DMF, 130 °C, 7 h, 99 %.

An alternative strategy was explored wherein the aldol condensation preceded the Mizoroki–Heck reaction. To this end, intermediate **4** was oxidatively cleaved to aldehyde **3** under Lemieux–Johnson conditions.^17^ TiCl_4_ mediated condensation led to the formation of the B-ring in 49 % yield over two steps. The resulting tetracycle **2** was subjected to the established Mizoroki–Heck reaction conditions, which we successfully applied in the synthesis of **12**. Clean formation of a single product was observed, but subsequent characterization by a number of methods including X-ray crystallographic analysis, revealed that the isomeric alkene (±)-**14**, which we have named *iso*-linoxepin, was formed (Scheme 6).

**Scheme 6:**
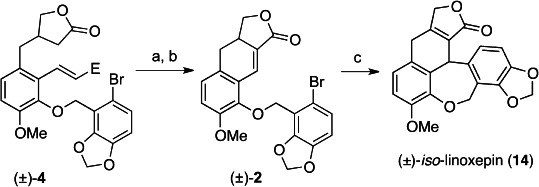
Undesired formation of (±)-*iso*-linoxepin (**14**). a) OsO_4_, NaIO_4_, TEBAC, THF/H_2_O 5:3, RT, 98 %; b) TiCl_4_, NEt_3_, CH_2_Cl_2_, −78 °C→−25 °C→RT, 3 h, 49 %; c) PdCl_2_ (20 mol %), PPh_3_ (44 mol %) NEt_3_ (10 equiv), microwave radiation, DMF, 130 °C, 7 h, 74 %; E=CO_2_*t*Bu, TEBAC=triethylbenzylammonium chloride.

We assume that the formation of undesired (±)-*iso*-linoxepin **14** arises from the elimination of a more accessible *syn* β-hydrogen under the reaction conditions (Scheme 7, pathway **C**). Conversely, this undesired process is attenuated by replacing NEt_3_ with a carboxylate base, allowing **1** to be obtained without **14** being formed. Although we do not know the exact origin of this change in selectivity, we can rationalize the result by formation of the Pd enolate so as to generate the diastereomeric intermediate **18**, which can undergo *syn*-β-hydride elimination (pathway **B**).^18^ Other mechanisms that achieve the same result include a C–H activation (pathway **A**)^8^, ^19^ or *anti*-β-hydride elimination.^20^

**Scheme 7:**
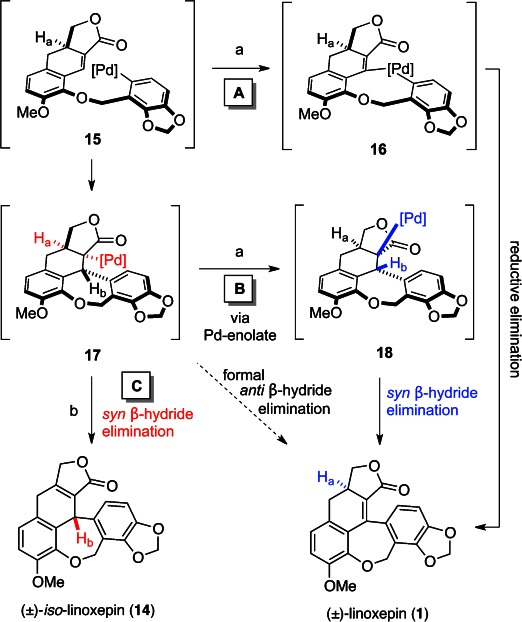
Mechanistic rationale for the formation of (±)-**1** and (±)-**14**:^21^ **15** was obtained by the oxidative addition of Pd^0^ to (±)-**2**; pathway **A**: C–H activation pathway by highly electrophilic Ar–Pd species; pathway **B**: Generation of a *syn* β-hydride (H_b_) by a Pd-enolate; Pathway **C**: Formation of (±)-*iso*-linoxepin **14** by a *syn* β-hydride elimination of H_a_; conditions: a) PdCl_2_ (20 mol %), PPh_3_ (44 mol %) NEt_3_ (10 equiv), microwave radiation, DMF, 130 °C, 7 h, 74 %; b) PdCl_2_ (20 mol %), PPh_3_ (44 mol %), CsOAc (10.0 equiv), DMF, 75 °C, 4 h, 78 %.

With an optimized route towards (±)-**1**, the stage was set for the enantiopure synthesis of (+)-**1**. Therefore (+)-**6** was prepared according the procedure of Zutter^12^ and subjected to the Catellani reaction. Optically active cinnamic acid derivative (−)-**4** was obtained with the same efficiency as its racemic counterpart. Although we were unable to determine the *ee* value of the product, conversion into dihydronaphthalene **2** provided an opportunity, and the *ee* was found to be 93 %. (+)-Linoxepin was directly formed by heating cyclization precursor (+)-**2** under Mizoroki–Heck conditions using cesium acetate as base (Scheme 8). These conditions resulted in the formation of the desired natural product (+)-linoxepin in 76 % yield. The partial loss of stereochemical information by the initial formation of *iso*-linoxepin (**14**) and subsequent base-mediated conversion into linoxepin (**1**) can be excluded. All attempts to convert (±)-**14** into its double-bond isomer **1** failed and resulted either in the recovery of unreacted starting material or in the formation of complex mixtures of highly fluorescent products.

**Scheme 8:**
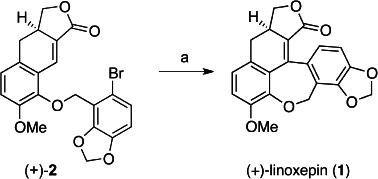
Synthesis of (+)-linoxepin (**1**). a) PdCl_2_ (20 mol %), PPh_3_ (44 mol %), CsOAc (10.0 equiv), DMF, 75 °C, 4 h, 76 %.

In conclusion, we have achieved the enantioselective, protecting-group-free, total synthesis of the challenging lignan (+)-linoxepin **1** using domino C–H functionalization with an overall yield of 30 %. This synthesis is the first reported application of the palladium-catalyzed Catellani reaction in the synthesis of a complex natural product. We note that the optical rotation of the synthetic material is higher (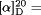
+90.0; *c*=0.25, CHCl_3_) than the reported value (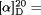
+23.0; *c*=0.93, CHCl_3_). All of the spectroscopic data of the final product are in complete agreement with the published data from the isolated material. It is noteworthy that Tietze and co-workers observed a higher optical rotation in their resolved material than was found in the isolated material (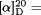
+96.1; *c*=0.61, CHCl_3_).^5^ X-ray crystallographic analysis unambiguously confirms the reported structure of linoxepin (**1**). We are continuing to investigate the origin of the change in regioselectivity in the final Mizoroki Heck reaction and will provide further details as they become available.

## Supplementary material

As a service to our authors and readers, this journal provides supporting information supplied by the authors. Such materials are peer reviewed and may be re-organized for online delivery, but are not copy-edited or typeset. Technical support issues arising from supporting information (other than missing files) should be addressed to the authors.
