# Supplementary material for: Total Synthesis of (+)-Linoxepin by Utilizing the Catellani Reaction
Source: Angew Chem Int Ed Engl. 2013 Apr 16;52(20):5305–8. doi: 10.1002/anie.201302327 (PMC3715096; doi:10.1002/anie.201302327)
Supplement: Supplementary file 1 [file anie0052-5305-SD1.pdf]

Angewandte Chemie
Eine Zeitschrift der Gesellschaft Deutscher Chemiker

Supporting Information

© Wiley-VCH 2013

69451 Weinheim, Germany

Total Synthesis of (+)-Linoxepin by Utilizing the Catellani Reaction**

*Harald Weinstabl, Marcel Suhartono, Zafar Qureshi, and Mark Lautens**

anie_201302327_sm_miscellaneous_information.pdf

General

Reactions

were conducted in flame-dried or oven-dried glassware under an atmosphere of dry argon. All reactions were first performed racemically, before being performed optically active. For racemic and optically active molecules, all spectroscopic data were in complete agreement. Therefore, the spectroscopic data for the enantiopure version is reported. Scale and yield of the reactions (racemic – enantiopure) is reported separately for each compound.

TLC

was performed with EMD TLC Silica gel 60 F254 aluminum sheets. Visualization was accomplished with 254 nm UV light followed by staining with potassium permanganate, anisaldehyde, phosphomolybdic acid or vanillin solution.

Flash and gradient column chromatography

was carried out using Silicycle Ultra-Pure 230–400 mesh silica gel.

Melting points

were taken on a Fisher-Johns melting point apparatus and are uncorrected.

IR spectra

were obtained using a Perkin-Elmer Spectrum 1000 FT-IR spectrometer as neat films or as solutions ($CHCl_3$ or CH_2Cl_2) on a NaCl plate. Data is presented as frequency of absorption (cm^{-1}).

^{1}H and ^{13}C NMR spectra

were recorded at 23 °C in $CDCl_3$ or DMSO-d_6 with a Bruker Avance 400 spectrometer or a Varian Mercury 400 spectrometer. Recorded shifts for protons are reported in parts per million (δ scale) and are referenced to residual proton signals in the NMR solvent ($CHCl_3$: δ = 7.26, DMSO-d_6: δ = 2.50). Chemical shifts for carbon resonances are reported in parts per million (δ scale) and are referenced to the carbon resonances of the solvent ($CDCl_3$: δ = 77.0, DMSO-d_6: δ = 39.43). Data are represented as follows: chemical shift, multiplicity (s=singlet, d=doublet, t=triplet, m=multiplet, b=broad), coupling constant (*J*, Hz) and integration. (* denotes rotamer peaks).

Proton and Carbon spectra (Linoxepin)

were recorded on a Agilent DD2 500 MHz spectrometer with an Agilent HC 5-mm XSens cryogenically-cooled probe. A ^{1}H pulse width of 45° was used, acquiring a spectral window of 7000 Hz (14 ppm) using 32k points. The ^{1}H 90° pulse width was 11.75 µs. A ^{13}C pulse width of 30° was used, acquiring a spectral window of 28750 Hz (230 ppm) using 64k points. The ^{13}C 90° pulse width was 21.4 µs. All pulse sequences used were provided by Agilent.
Funded by the Canadian Foundation for Innovation, project number 19119, and the Ontario MRI.

Crystallographic analysis

Crystal structures were obtained on a Bruker Kappa APEX-DUO CCD equipped with a Bruker Triumph or multi-layer optics monochromator using $Mo_{K\alpha}$ or $Cu_{K\alpha}$ irradiation. Data collection was performed using Bruker APEX2 software, data reduction using Bruker SAINT, and structure solution using Sheldrick's SHELXS-97.
CCDC 929322 – 929326 contains the supplementary crystallographic data for this paper. These data can be obtained free of charge from the Cambridge Crystallographic Data Centre via http://www.ccdc.cam.ac.uk/data_request/cif.

High resolution mass spectra

were obtained from a SI2 Micromass 70S- 250 mass spectrometer (EI), an ABI/Sciex Qstar mass spectrometer (ESI) with an Ionics HSID interface, or a JEOL AccuTOF model JMS-T1000LC mass spectrometer equipped with a IONICS® Direct Analysis in Real Time (DART) ion source.

Chemicals

were purchased from Sigma Aldrich Co. LLC, Alfa Aesar, or Combi-Blocks and used without further purification unless otherwise stated.

Solvents

used in reactions were purified before use. Tetrahydrofuran was distilled from molten sodium metal. Dichloromethane (DCM), acetonitrile, NEt_3 and pyridine were distilled from CaH_2. Acetone was distilled over Drierite® (anhydrous $CaSO_4$). DMF (anhydrous grade) was purchased from Sigma Aldrich.

Experimental procedures

2-(2-methoxyphenoxy)oxane (18)

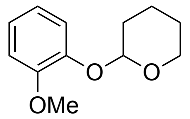

To a solution of guaiacol (10.0 g, 80.55 mmol, 1.0 equiv.) in DCM (70 mL) was added 3,4-dihydro-2*H*-pyran (67.8 g, 73.0 mL, 805.5 mmol, 10 equiv.) and PPTS (200 mg, 8.05 mmol, 0.1 equiv.). The reaction was stirred for 4 h at RT and which point saturated NaOH (20 mL) was added. The reaction mixture was extracted with DCM and the combined organic phases were dried over $MgSO_4$ and concentrated *in vacuo*. Purification by flash column chromatography (hexanes/EtOAc 10:1) yielded acetal **18** as a colourless oil (15.95 g, 95%).

R_f: 0.55 (hexanes/EtOAc, 3:1).

^{1}H NMR (400 MHz, DMSO-d_6): δ = 7.07 (dd, *J* = 8, 1.6, 1H), 7.00-6.93 (m, 2H), 6.85 (m, 1H), 5.36 (t, *J* = 3.2 Hz, 1H), 3.84 (m, 1H), 3.76 (s, 3H), 3.43 (m, 1H), 1.87 (m, 1H), 1.78-1.75 (m, 2H), 1.62-1.52 (m, 3H).

^{13}C NMR (100 MHz, DMSO-d_6): δ = 149.9, 145.6, 122.3, 120.5, 117.6, 112.6, 96.6, 61.3, 55.5, 29.8, 24.7, 18.5.

IR (neat) ν_{max} = 3063, 2943, 2874, 2843, 2361, 1593, 1501, 1454, 1358, 1254, 1211, 1115, 1026, 961, 914, 745.

HRMS (EI) found: $[M]^+$ *m/z* = 208.1098, calcd. for $C_{12}H_{16}O_3$: 208.1099

2-Iodo-6-methoxyphenol (11)

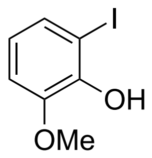

To a solution of acetal **18** (4.00 g, 19.21 mmol, 1.0 equiv.) in THF (40 mL) at 0 °C was added *n*BuLi (1.70 M in *n*-hexane, 17.0 mL, 28.82 mmol, 1.5 equiv.) dropwise. The reaction was allowed to warm to room temperature and stirred under Ar for 3 h at which point it was cooled to -50 °C and I_2 (9.75 g, 38.42 mmol, 2.0 equiv.) dissolved in THF (60 mL) was added dropwise. After warming to room temperature overnight the reaction mixture was concentrated *in vacuo* and the residue was suspended in water and extracted with DCM. The combined organic phases were washed with saturated Na_2SO_3 solution and then water. After drying over $MgSO_4$ and concentration under reduced pressure, the residue was adsorbed on silica. Purification by column chromatography (hexanes/EtOAc 3:1) yielded **11** as a white solid (4.70 g, 98%). A further purification by recrystallization (DCM /hexanes) gave white crystals.

R_f: 0.4 (hexanes/EtOAc 3:1).

Melting Point: 47-49 °C.

^{1}H NMR (400 MHz, $CDCl_3$): δ = 7.29 (dd, *J* = 8, 1.2, 1H), 6.83 (dd, *J* = 8, 1.2, 1H), 6.63 (t, *J* = 8, 1H), 6.08 (s, 1H), 3.89 (s, 3H).

^{13}C NMR (100 MHz, $CDCl_3$): δ = 146.3, 145.8, 130.7, 121.6, 110.7, 81.4, 56.2.

IR (neat) ν_{max} = 3480, 2365, 1589, 1474, 1435, 1346, 1277, 1223, 1126, 1022, 799, 756, 714.

HRMS (EI) found: $[M]^+$ *m/z* = 249.9485, calcd. for $C_7H_7IO_2$: 249.9491

5-Bromo-2*H*-1,3-benzodioxole-4-carbaldehyde (19)

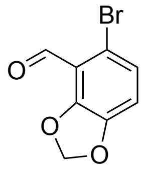

To a solution of diisopropylamine (3.15 g, 4.37 mL, 31.13 mmol, 1.25 equiv.) in THF (60 mL) at -78 °C was added *n*BuLi (1.8 M in *n*-hexane, 17.3 mL, 31.13 mmol, 1.25 equiv.) dropwise. After 15 min 5-Bromo-2*H*-1,3-benzodioxole (**8**) (5.00 g, 3.00 mL, 24.90 mmol, 1.0 equiv.) was added and reaction was allowed to stir -78 °C for 1h. DMF (4.37 g, 4.6 mL, 59.8 mmol, 2.0 equiv.) was added dropwise to the reaction mixture, which was then allowed to warm to RT overnight. The reaction was quenched with saturated NH_4Cl, and extracted with EtOAc. The combined organic phases were concentrated without drying and purified by recrystallization from DCM/hexanes to give aldehyde **19** as yellow crystals (5.42 g, 95%).

Melting Point: 163-165 °C.

^{1}H NMR (400 MHz, $CDCl_3$): δ = 10.29 (s, 1H), 7.11 (d, *J* = 8.4, 1H), 6.85 (dd, J=8.4, 1H), 6.16 (s, 2H).

^{13}C NMR (100 MHz, $CDCl_3$): δ = 190.2, 149.5, 148.7, 126.2, 117.5, 115.5, 113.5, 103.3.

IR (neat) ν_{max} = 1678, 1616, 1582, 1451, 1397, 1242, 1207, 1115, 1045, 1015, 876, 806.

See: Sit *et al.*, *J. Med. Chem*. **2002**, *45*, 3660-3668

(5-Bromo-2*H*-1,3-benzodioxol-4-yl)methanol (9)

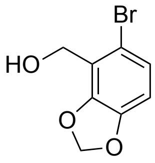

To a solution of 5-Bromo-2*H*-1,3-benzodioxole-4-carbaldehyde (**19**, 500 mg, 2.18 mmol, 1.0 equiv.) in THF (20 mL) was added powdered $NaBH_4$ (82.59 mg, 2.18 mmol, 1.0 equiv.) in small portions. The reaction was refluxed for 90 min then quenched with water and then acidified by the addition of 0.5M $KHSO_4$. The aqueous layer was extracted three times with EtOAc. The combined organic phases were dried over $MgSO_4$ and concentrated *in vacuo*. After purification by column chromatography (hexanes/ethyl acetate 3:1) benzyl alcohol **9** was obtained as a colourless solid (486 mg, 96%).

Rf: 0.15 (hexanes/EtOAc, 3:1).

Melting Point: 93-94 °C

^{1}**H NMR** (400 MHz, $CDCl_3$): δ = 7.04 (d, *J* = 8.4, 1H), 6.65 (d, *J* = 8.4, 1H), 6.02 (s, 2H,), 4.76 (d, *J* = 6.8, 2H), 2.13 (t, *J* = 6.8, 1H).

^{13}**C NMR** (100 MHz, $CDCl_3$): δ = 147.18, 147.15, 125.3, 121.7, 115.0, 109.2, 101.9, 59.5.

IR (neat) ν_{max} = 3356, 1439, 1242, 995, 926, 775.

HRMS (ESI) found: $[M+Na]^+$ *m/z* = 252.9476, calcd. for $C_8H_7BrNaO_3$: 252.9470

5-Bromo-(4-iodomethyl)-2*H*-1,3-benzodioxole (10)

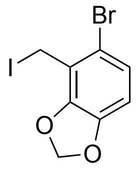

To a solution of (5-Bromo-2*H*-1,3-benzodioxol-4-yl)methanol (**9**, 5.00 g, 21.64 mmol, 1.0 equiv.) in MeCN (30 mL) was added TMSCl (4.71 g, 5.55 mL, 43.28 mmol, 2.0 equiv.) and NaI (6.49 g, 43.28 mmol, 2.0 equiv.) successively. The reaction mixture was stirred for 1 h at RT. During that time it turned brown. The mixture was quenched with saturated Na_2SO_3 solution and extracted twice with DCM. The combined organic phases were dried over $MgSO_4$ and concentrated *in vacuo*. After the purification by column chromatography (hexanes/EtOAc 25:1) benzyl iodide **10** was obtained as a colourless solid (7.30 g, 99%) was obtained.

Rf: 0.45 (hexanes / EtOAc).

Melting Point: 79 – 80 °C.

^{1}H NMR (400 MHz, $CDCl_3$): δ = 7.01 (d, *J* = 8.4, 1H), 6.62 (d, *J* = 8.4, 1H), 6.06 (s, 2H,), 4.46 (s, 2H).

^{13}C NMR (100 MHz, $CDCl_3$): δ = 147.1, 146.6, 125.6, 120.5, 115.1, 109.1, 102.3, -2.3.

IR (neat) ν_{max} = 2905, 1493, 1458, 1420, 1250, 1215, 1126, 1049, 999, 922, 876, 810, 664.

HRMS (EI) found: $[M]^+$ *m/z* = 339.8584, calcd. for $C_8H_6BrIO_2$: 339.8596

5-Bromo-4-(2-iodo-6-methoxyphenoxymethyl)-2*H*-1,3-benzodioxole (5)

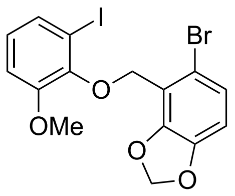

2-Iodo-6-methoxyphenol (**11**, 6.31 g, 25.22 mmol, 1.0 equiv.) and 5-bromo-(4-iodomethyl)-2*H*-1,3-benzodioxole (**10**, 9.03 g, 26.48 mmol, 1.05 equiv.) were dissolved in anhydrous acetone (200 mL). K_2CO_3 (10.46 g, 75.66 mmol, 3.0 equiv.) was added and the reaction mixture was refluxed for 2 h. For workup water (50 mL) was added and the solution was extracted with DCM. The combined organic phases were dried over $MgSO_4$ and concentrated under reduced pressure. The residue was purified by column chromatography (hexanes/EtOAc 10:1). For further purification the resulting colourless solid was recrystallized from hexanes/EtOAc at -25 °C to yield ether **5** as white crystals.

Yield: 10.95 g (94%).

Rf: 0.55 (hexanes/EtOAc, 3:1).

Melting point: 61 – 63 °C.

^{1}H NMR (400 MHz, $CHCl_3$) δ = 7.32 (dd, *J* = 8.0, 1.5 Hz, 1H), 7.04 (d, *J* = 8.2 Hz, 1H), 6.87 (dd, *J* = 8.1, 1.5 Hz, 1H), 6.79 (d, *J* = 8.0 Hz, 1H), 6.67 (d, *J* = 8.3 Hz, 1H), 5.95 (s, 2H), 5.21 (s, 2H), 3.82 (s, 3H), 2.17 (s, 1H).

^{13}C NMR (100 MHz, $CDCl_3$): δ = 153.0, 148.6, 147.9, 146.9, 130.7, 125.9, 125.1, 118.7, 116.8, 112.7, 109.6, 101.8, 92.9, 68.3, 56.0.

IR (neat) ν_{max} = 2959, 2936, 2893, 1578, 1454, 1258, 1219, 1030, 961, 802, 764.

HRMS (ESI) found: $[M+Na]^+$ *m/z* = 484.8852, calcd. for $C_{15}H_{12}BrINaO_4$: 484.8861

(+)-(*R*)-4-(iodomethyl)dihydrofuran-2(3*H*)-one (6)

Was synthesized according to
J.-M. Adam, J. Foricher, S. Hanlon, B. Lohri, G. Moine, R. Schmid, H. Stahr, M. Weber, B. Wirz, U. Zutter, *Org. Process Res. Dev.* **2011**, *15*, 515–526.

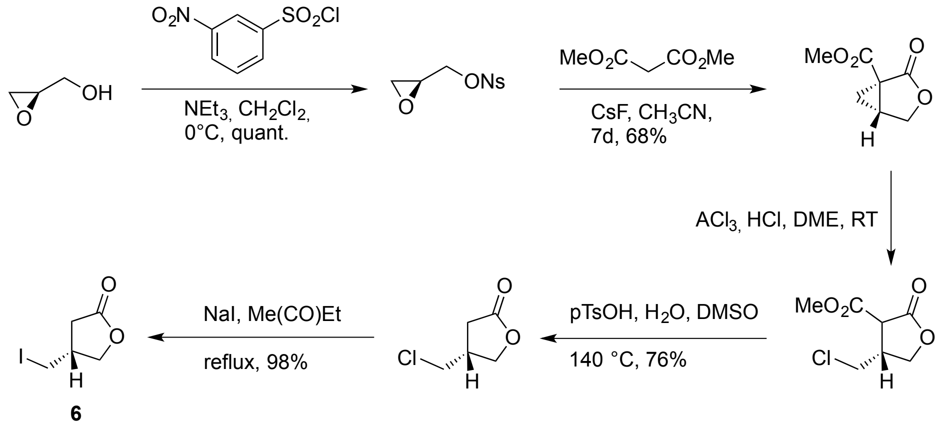

Data for compound **6**

^{1}H NMR (400 MHz, $CDCl_3$) δ = 4.44 (dd, *J* = 9.4, 7.4 Hz, 1H), 4.03 (dd, *J* = 9.4, 6.6 Hz, 1H), 3.34 - 3.09 (m, 2H), 2.96 - 2.79 (m, 1H), 2.70 (dd, *J* = 17.6, 8.6 Hz, 1H), 2.35 (dd, *J* = 17.7, 7.5 Hz, 1H)

^{13}C NMR (100 MHz, $CDCl_3$) δ = 175.44, 73.16, 37.67, 35.68, 6.06

HRMS (DART) found: $[M+H]^+$ *m/z* = 226.95682, calcd. for $C_5H_8IO_2$: 226.95690

Optical rotation: $[\alpha]_D^{20}$: +33.8 (c = 1.1, $CHCl_3$).

(±)-4-(iodomethyl)dihydrofuran-2(3*H*)-one (6)

Was synthesized according to
J.-M. Adam, J. Foricher, S. Hanlon, B. Lohri, G. Moine, R. Schmid, H. Stahr, M. Weber, B. Wirz, U. Zutter, *Org. Process Res. Dev.* **2011**, *15*, 515–526.

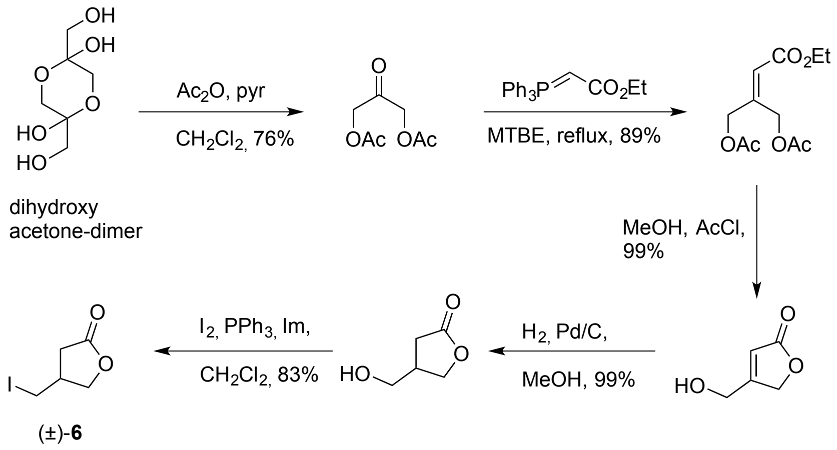

All spectroscopic data were in full agreement with the reported literature values.

(–)-tert-butyl (*R*,*E*)-3-(2-((5-bromobenzo[*d*][1,3]dioxol-4-yl)methoxy)-3-methoxy-6-((5-oxotetrahydrofuran-3-yl)methyl)phenyl)acrylate (4)

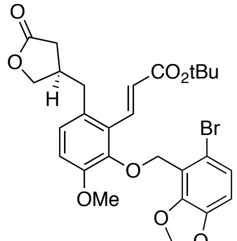

4

$Pd(OAc)_2$ (38.5 mg, 0.17 mmol, 0.10 equiv.), PPh_3 (99.1 mg, 0.38 mmol, 0.22 equiv.), Cs_2CO_3 (2.80 g, 8.58 mmol, 5.0 equiv.), 5-Bromo-4-(2-iodo-6-methoxyphenoxymethyl)-2*H*-1,3-benzodioxole (**5**) (797 mg, 1.72 mmol, 1.0 equiv.), tert-butyl acrylate (1.26 mL, 8.58 mmol, 5.0 equiv.) and iodolactone (+)-**6** (1.94 g, 8.58 mmol, 5.0 equiv.) were added successively to a sealable pressure flask under Ar. Degassed, anhydrous DMF (2 mL) was added and the reaction mixture was allowed to stir at RT while Ar was bubbled through the heterogeneous mixture. Norbornene (808.2 mg, 8.58 mmol, 5.0 equiv.) was added and the flask was again purged with Ar before being sealed and heated at 90 °C for 5h. The reaction was cooled to RT and quenched by the addition of sat. aq. NH_4Cl solution. Water and a 1/1 mixture of EtOAc/hexanes were added and the phases were separated. The organic layer was dried with brine and $MgSO_4$ and the solvents were removed under reduced pressure. The crude material was purified by silica gel column chromatography (hexanes:EtOAc 3:1 → 2:1) to give lactone (–)-**4**. After recrystallization from EtOAc / hexanes a white solid (860 mg, 1.53 mmol, 89%) was obtained.

^{1}H NMR (400 MHz, $CDCl_3$): δ = 7.42 (d, *J* = 16.1 Hz, 1H), 6.99 (d, *J* = 8.2 Hz, 1H), 6.84 (s, 2H), 6.64 (d, *J* = 8.2 Hz, 1H), 6.42 (d, *J* = 16.1 Hz, 1H), 5.95 (s, 2H), 5.11 (s, 2H), 4.25 (dd, *J* = 9.2, 6.6 Hz, 1H), 3.96 (dd, *J* = 9.2, 5.9 Hz, 1H), 3.88 (s, 3H), 2.82 – 2.66 (m, 3H), 2.52 (dd, *J* = 17.4, 7.7 Hz, 1H), 2.22 (dd, *J* = 17.5, 6.8 Hz, 1H), 1.50 (s, 9H).

^{13}C NMR (100 MHz, $CDCl_3$): δ = 176.67, 166.38, 152.19, 148.33, 146.93, 146.89, 136.32, 129.92, 128.76, 126.85, 125.72, 125.05, 118.62, 116.39, 112.68, 109.60, 101.97, 80.34, 72.44, 68.29, 55.96, 36.46, 36.21, 34.13, 28.19.

IR (neat) ν_{max} = 3434, 2977, 1780, 1767, 1696, 1627, 1575, 1480, 1456, 1368, 1276, 1262, 1154, 1052, 1022, 764, 749.

HRMS (ESI) found: $[M+NH_4]^+$ m/z = 578.13928, calcd. for $C_{27}H_{33}BrNO_8$: 578.13895.

Melting point: 113 – 116 °C

Optical rotation: $[\alpha]_D^{20}$: -4.1 (c = 0.29, $CHCl_3$)

Scale of the racemic experiment: limiting compound **5** (6.34 g, 13.1 mmol, 1.0 equiv.); yield: 92% (6.77 g, 12.0 mmol)

(–)-(*R*)-2-((5-bromobenzo[*d*][1,3]dioxol-4-yl)methoxy)-3-methoxy-6-((5-oxotetrahydrofuran-3-yl)methyl)benzaldehyde (3)

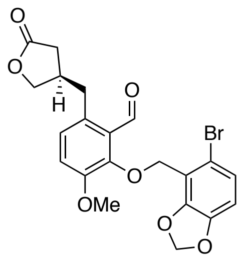

3

Catellani product **4** (420 mg, 0.75 mmol, 1.0 equiv.) was dissolved in THF (5 mL) and water (3 mL) was added. The reaction mixture was cool to 0 °C when $NaIO_4$ (480 mg, 2.25 mmol, 3.0 equiv), triethylbenzylammonium chloride (TEBAC, 17.1 mg, 0.08 mmol, 0.1 equiv.), and a small grain of OsO_4 (catalytic amount, CAUTION, highly toxic) were added. The reaction was allowed to stir 10 minutes at this temperature then the ice bath was removed and the reaction was allowed to room temperature. Stirring was continued for 5 hours before the reaction was quenched by the addition of sat. aq. Na_2SO_3 solution. EtOAc was added and the phases were separated. The aqueous solution was extracted with EtOAc an additional two times. The organic layers were combined and dried with $MgSO_4$. The solvent was removed *in vacuo* and the product was purified by silica gel chromatography (hexanes/EtOAc 4:1 → 3:1) to yield aldehyde (–)-**3** (347mg, 0.75 mmol, 99%) as white crystals.

^{1}H NMR (400 MHz, $CDCl_3$) δ = 10.38 (d, *J* = 0.7 Hz, 1H), 7.08 (d, *J* = 8.4 Hz, 1H), 7.04 (d, *J* = 8.3 Hz, 1H), 6.87 (d, *J* = 8.3 Hz, 1H), 6.69 (d, *J* = 8.3 Hz, 1H), 5.95 (s, 2H), 5.32 (s, 2H), 4.28 (dd, *J* = 9.1, 7.0 Hz, 1H), 3.99 (dd, *J* = 9.1, 6.3 Hz, 1H), 3.95 (s, 3H), 3.05 – 2.89 (m, 2H), 2.85 2.65 (m, 1H), 2.48 (dd, *J* = 17.4, 8.2 Hz, 1H), 2.23 (dd, *J* = 17.4, 7.2 Hz, 1H).

^{13}C NMR (100 MHz, $CDCl_3$) δ = 193.03, 177.20, 152.71, 152.06, 148.45, 147.05, 131.82, 128.26, 127.06, 125.46, 117.56, 117.18, 116.29, 110.18, 102.02, 72.82, 69.33, 56.13, 36.62, 36.25, 34.08.

IR (neat) ν_{max} = 2917, 1775, 1730, 1684, 1574, 1488, 1457, 1401, 1369, 1257, 1226, 1169, 1052, 1021, 933, 853, 803

Melting point: 110 – 112 °C

HRMS (DART) found: $[M+H]^+$ *m/z* = 463.03942, calcd. for $C_{21}H_{20}BrO_7$: 463.03924

Optical rotation: $[\alpha]_D^{20}$ = -2.4 (c = 1.13, $CHCl_3$)

HPLC analysis: enantiomers not separable, Chiralpak® AD-H, 70/30 hexanes/IPA, 1 mL / min, t_{R1} = 10.60 min, t_{R2} = not detectable

Scale of the racemic experiment: limiting compound **4** (6.70 g, 11.9 mmol, 1.0 equiv.); yield: 98% (5.41 g, 11.7 mmol)

(+)-(*R*)-8-((5-bromobenzo[*d*][1,3]dioxol-4-yl)methoxy)-7-methoxy-3a,4-dihydronaphtho[2,3-*c*]furan-1(3*H*)-one (2)

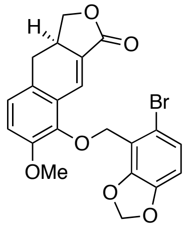

2

Aldehyde **3** (110 mg, 0.24 mmol, 1.equiv.) was dissolved in anhydrous CH_2Cl_2 (8 ml) and the homogenous solution was cooled to -78 °C. $TiCl_4$ (29 µL, 0.26 mmol, 1.1equiv.) and NEt_3 (83 µL, 0.60 mmol, 2.5 equiv.) were added and the solution was allowed to warm to -25 °C were stirring was continued for 3 hours. Now additional $TiCl_4$ (29 µL, 0.26 mmol, 1.1equiv.) and NEt_3 (83 µL, 0.60 mmol, 2.5 equiv) were added and the reaction mixture was allowed to warm to RT within 1 hour before it was quenched by the addition of sat. aq. $NaHCO_3$. The crude biphasic mixture was filtered over a short plug of Celite® and repeatedly washed with CH_2Cl_2. The phases were separated and the organic phase was dried with $MgSO_4$. Solvents were removed under reduced pressure and the crude material was purified using column chromatography (hexanes/EtOAc 4:1 → 3:1). Dihydronaphthalene **2** (56 mg, 0.23 mmol, 53%) was obtained as white solid.

^{1}H NMR (400 MHz, $CHCl_3$) δ = 7.84 (d, *J* = 3.2 Hz, 1H), 6.99 (d, *J* = 8.3 Hz, 1H), 6.92 (d, *J* = 8.2 Hz, 1H), 6.85 (d, *J* = 8.2 Hz, 1H), 6.64 (d, *J* = 8.2 Hz, 1H), 6.10 (dd, *J* = 27.2, 1.4 Hz, 2H), 5.22 (d, *J* = 0.7 Hz, 2H), 4.71 (td, *J* = 8.7, 0.6 Hz, 1H), 3.97 (t, *J* = 8.8 Hz, 1H), 3.91 (s, 3H), 3.20 3.02 (m, 1H), 2.94 (dd, *J* = 14.9, 6.8 Hz, 1H), 2.60 (td, *J* = 15.3, 1.3 Hz, 1H).

^{13}C NMR (100 MHz, $CDCl_3$) δ = 169.66, 152.22, 148.57, 147.16, 145.70, 128.56, 127.60, 127.26, 127.16, 125.13, 123.68, 118.11, 116.27, 113.44, 109.78, 102.30, 72.49, 68.81, 56.13, 34.43, 32.29.

IR (neat) ν_{max} = 3390, 2897, 1750, 1662, 1572, 1483, 1456, 1336, 1260, 1242, 1199, 1182, 1121, 1093, 1052, 1037, 999.

HRMS (DART) found: $[M+H]^+$ *m/z* = 445.02720, calcd. for $C_{21}H_{18}BrO_6$: 445.02868

Optical rotation: $[\alpha]_D^{20}$ = + 103.3 (c = 1.05, $CHCl_3$)

Melting point: 198 – 201 °C

HPLC analysis: Chiralpak® AD-H, 70/30 hexanes/IPA, 1 mL / min, t_{R1} = 10.51 min, t_{R2} = 12.79 min, 93% ee

Scale of the racemic experiment: limiting compound **3** (1.38 g, 3.0 mmol, 1.0 equiv.); yield: 49% (650 mg, 1.5 mmol)

(+)-linoxepin (1) or
(*R*)-6-methoxy-9a,10-dihydro-4*H*-[1,3]dioxolo[4',5':3,4]benzo[1,2-e]furo[3',4':6,7]naphtho[1,8-*bc*]oxepin-12(9*H*)-one

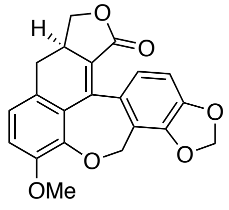

(+)-Linoxepin (**1**)

A flame dried sealable vial was charged with dihydronaphthalene **2** (10.0 mg, 21.8 µmmol, 1.0 equiv.), $PdCl_2$ (0.77 mg, 4.4 µmol, 0.2 equiv.), PPh_3 (2.5 mg, 9.6 µmol, 0.44 equiv.), and anhydrous CsOAc (41.8 mg, 218 µmol, 10.0 equiv.). The vial was flushed with Ar for 2 minutes before degassed, anhydrous DMF (1 mL) was added. The suspension was stirred for 5 min at RT and purged with Ar. The vial was sealed and heated to 75°C for four hours. The reaction was quenched by the addition of sat. aq. NH_4Cl solution and the aqueous layer was extracted two times with a 1/1 mixture of EtOAc/hexanes. The combined organic layers were dried over $MgSO_4$ and the solvents were removed under reduced pressure. The title compound was obtained as a yellow solid (6.0 mg, 16.5 µmol, 76%) after column chromatography using silica gel (hexanes/EtOAc 2:1).

^{1}H NMR (500 MHz, $CDCl_3$): δ = 6.87 (d, *J* = 8.0 Hz, 1H), 6.84 (dd, *J* = 8.2, 1.2 Hz, 1H), 6.80 (d, *J* = 8.1 Hz, 1H), 6.74 (d, *J* = 8.0 Hz, 1H), 6.04 – 6.02 (m, 2H), 5.39 (dd, *J* = 12.6, 1 Hz, 1H), 5.14 (d, *J* = 12.5 Hz, 1H), 4.68 (t, *J* = 8.9 Hz, 1H), 4.03 (t, *J* = 8.7 Hz, 1H), 3.85 (s, 3H), 3.36 – 3.16 (m, 1H), 2.99 (dd, *J* = 14.7, 5.7 Hz, 1H), 2.66 (td, *J* = 14.8, 1.3 Hz, 1H);

^{13}C NMR (125 MHz, $CDCl_3$): δ = 168.83, 149.43, 149.04, 148.52, 145.68, 144.79, 129.43, 128.15, 124.35, 124.14, 122.22, 119.82, 116.50, 111.83, 108.12, 101.85, 70.00, 64.66, 56.18, 36.84, 34.46;

IR (neat) ν_{max} = 2900, 1748, 1661, 1572, 1481, 1464, 1436, 1300, 1277, 1264, 1244, 1199, 1183, 1102, 1032, 1013, 913, 760

HRMS (DART) [M+H]+ m/z = 365.10195 calcd. for $C_{21}H_{17}O_6$: 365.10251.

Melting point: decomp. 228 °C

Optical rotation: $[\alpha]_D^{20}$: + 90.0 (c = 0.25, $CHCl_3$).

Scale of the racemic experiment: limiting compound **2** (223 mg, 0.5 mmol, 1.0 equiv.); yield: 78% (142 mg, 0.39 mmol)

(±)–*tert*-butyl (*Z*)-2-(10-methoxy-7-((5-oxotetrahydrofuran-3-yl)methyl)-[1,3]dioxolo[4',5':3,4]benzo[1,2-*e*]benzo[*b*]oxepin-6(12*H*)-ylidene)acetate (12)

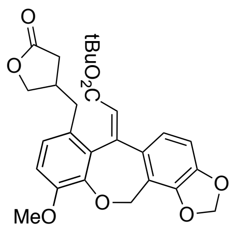

12

In a sealable tube $PdCl_2$ (3.16 mg, 0.018 mmol, 0.2 equiv.) and PPh_3 (10.3 mg, 0.039 mmol, 0.44 mmol) were suspended in dry DMF (1.5 mL). The mixture was stirred under argon for 10 min at RT. Then *tert*-butyl (*R,E*)-3-(2-((5-bromobenzo[*d*][1,3]dioxol-4-yl)methoxy)-3-methoxy-6-((5-oxotetrahydrofuran-3-yl)methyl)phenyl)acrylate (**4**) (50 mg, 0.089 mmol, 1.0 equiv.) and dry NEt_3 (126 µL, 0.89 mmol, 10 equiv.) were added into the tube. Before the tube was sealed, the content was flushed with argon for additional five minutes. The reaction mixture was heated in the microwave for 7 h at 130 °C. For workup the reaction mixture was diluted with water and extracted with EtOAc. The combined organic phases were dried over $MgSO_4$ and concentrated to dryness *in vacuo*. The dark residue was purified by column chromatography (hexanes/EtOAc 4:1 → 3:1) to obtain tetracycle **12** (42 mg, 0.089 mmol, 99%) as off white solid.

^{1}H NMR (400 MHz, $CDCl_3$) δ = 6.97 6.77 (m, 3H), 6.71 (dd, *J* = 8.0, 3.1 Hz, 1H), 6.21 (s, 1H), 5.98 (d, *J* = 1.38 Hz, 1H), 5.92 (bs, 1H), 5.34 (d, *J* = 16.3 Hz, 1H), 4.90 (dd, *J* = 16.2, 1.2 Hz, 1H), 4.11 (td, *J* = 6.8, 2.3 Hz, 1H), 3.91 (m, 0.5H)*, 3.88 (s, 3H), 3.69 (dd, *J* = 9.2, 5.8 Hz, 0.5H)*, 2.78 (dt, *J* = 13.9, 7.0 Hz, 1H), 2.63 (dt, *J* = 14.6, 7.4 Hz, 1H), 2.53 (ddd, *J* = 13.7, 7.7, 1.8 Hz, 1H), 2.42 (ddd, *J* = 17.5, 8.1, 5.7 Hz, 1H), 2.16 (dd, *J* = 17.5, 6.6 Hz, 0.5H)*, 2.00 (dd, *J* = 17.5, 6.8 Hz, 0.5H)*, 1.29 (s, 9H).

stars (*) indicate rotamer peaks

^{13}C NMR (100 MHz, $CDCl_3$) δ = 176.80, 176.67, 164.49, 164.47, 150.60, 150.55, 150.40, 150.37, 147.71, 147.70, 143.85, 143.83, 142.78, 142.69, 136.76, 136.71, 131.79, 131.75, 127.71, 127.62, 125.39, 125.29, 123.21, 123.15, 121.12, 121.09, 118.53, 118.51, 111.71, 111.64, 107.13, 107.06, 101.89, 80.44, 72.78, 72.60, 68.27, 55.98, 36.49, 36.25, 35.38, 35.28, 34.43, 34.32, 27.82.

The occurrence of rotamers caused a duplication of signals

IR (neat) ν_{max} = 2975, 2916, 1779, 1771, 1712, 1693, 1493, 1471, 1456, 1368, 1253, 1164, 1149, 1043, 1019

Melting point: 212 – 214 °C

HRMS (DART) found: $[M+NH_4]^+$ *m/z* = 498.21393, calcd. for $C_{27}H_{32}NO_8$: 498.21279

(±)–Isolinoxepin or
6-methoxy-10,12b-dihydro-4*H*-[1,3]dioxolo[4',5':3,4]benzo[1,2-*e*]furo[3',4':6,7]naphtho[1,8-*bc*]oxepin-12(9*H*)-one (14)

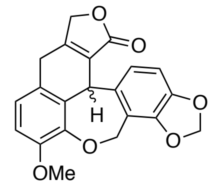

(±)-*iso*-linoxepin (**14**)

In a sealable tube $PdCl_2$ (0.52 mg, 0.003 mmol, 0.2 equiv.) and PPh_3 (1.7 mg, 0.006 mmol, 0.44 mmol) were suspended in dry DMF (0.5 mL). The mixture was stirred under argon for 10 min at RT. Then 8-((5-bromobenzo[*d*][1,3]dioxol-4-yl)methoxy)-7-methoxy-3a,4-dihydronaphtho[2,3-*c*]furan-1(3*H*)-one (**3**) (6.5 mg, 0.015 mmol, 1.0 equiv.) and dry NEt_3 (20 µL, 0.146 mmol, 10 equiv.) were added into the tube. Before the tube was sealed, the content was flushed with argon for additional five minutes. The reaction mixture was heated in the microwave for 7 h at 130 °C. For workup the reaction mixture was diluted with water and extracted with EtOAc. The combined organic layers were dried over $MgSO_4$ and concentrated to dryness *in vacuo*. The dark residue was purified by column chromatography (hexanes/EtOAc 2:1) to obtain *iso*-Linoxepin **14** (4 mg, 0.011 mmol, 74%) as off white solid.

^{1}H NMR (400 MHz, $CDCl_3$) δ = 6.90 (d, *J* = 8.5 Hz, 1H), 6.84 (d, *J* = 8.5 Hz, 1H), 6.53 (d, *J* = 8.1 Hz, 1H), 6.25 (d, *J* = 8.2 Hz, 1H), 5.89 (dd, *J* = 24.7, 1.4 Hz, 2H), 5.80 (bs, 1H), 5.59 (d, *J* = 15.5 Hz, 1H), 5.18 (d, *J* = 15.6 Hz, 1H), 5.10 (d, *J* = 17.2 Hz, 1H), 4.93 (dd, *J* = 17.3, 2.3 Hz, 1H), 3.88 (s, 3H), 3.76 (dd, *J* = 22.0, 4.1 Hz, 1H), 3.61 (dd, *J* = 22.1, 3.1 Hz, 1H).

^{13}C NMR (100 MHz, $CDCl_3$) δ = 172.80, 161.64, 151.00, 146.46, 144.65, 143.73, 134.25, 131.49, 126.30, 123.98, 121.72, 117.76, 117.04, 111.48, 106.29, 101.50, 71.70, 67.70, 56.09, 34.02, 28.07.

HRMS (ESI) found: $[M+Na]^+$ *m/z* = 387.0852, calcd. for $C_{21}H_{16}NaO_6$: 387.0839

HPLC traces

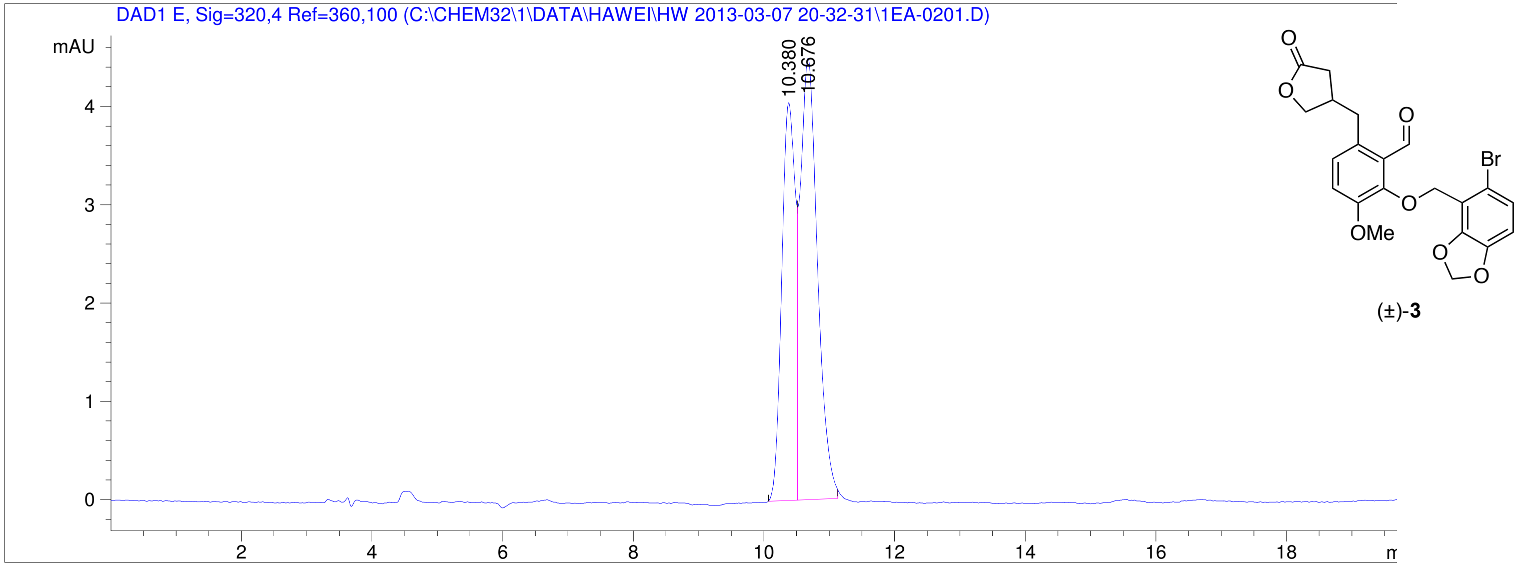

(±)-**3**

```
=====================================================================
                         Area Percent Report
=====================================================================

Sorted By             :      Signal
Multiplier            :      1.0000
Dilution              :      1.0000
Use Multiplier & Dilution Factor with ISTDs


Signal 1: DAD1 E, Sig=320,4 Ref=360,100

Peak RetTime Type  Width     Area      Height     Area
  #   [min]        [min]   [mAU*s]     [mAU]        %
----|-------|----|-------|----------|----------|--------|
   1  10.380 BV    0.2189   58.56232    4.04560  41.6701
   2  10.676 VB    0.2669   81.97572    4.49119  58.3299

Totals :                   140.53804    8.53679
```

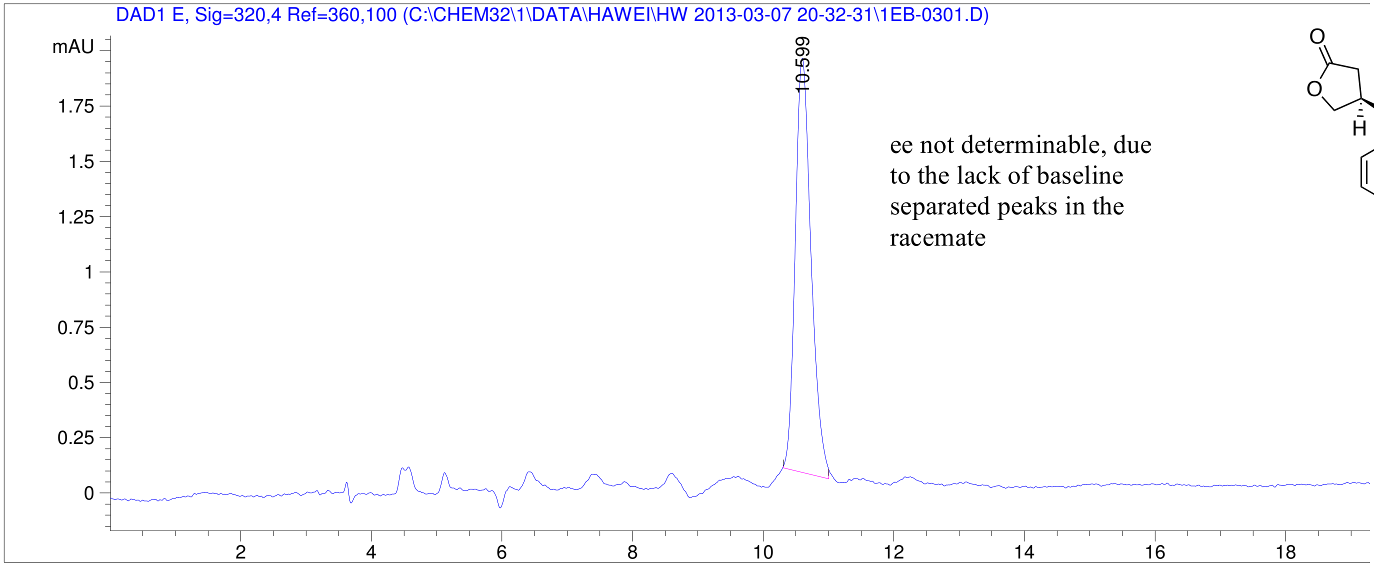

ee not determinable, due to the lack of baseline separated peaks in the racemate

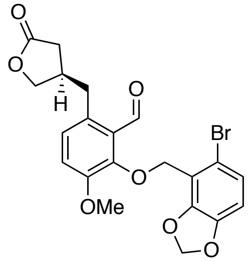

(–)-**3**

```
=====================================================================
                         Area Percent Report
=====================================================================

Sorted By             :      Signal
Multiplier            :      1.0000
Dilution              :      1.0000
Use Multiplier & Dilution Factor with ISTDs


Signal 1: DAD1 E, Sig=320,4 Ref=360,100

Peak RetTime Type  Width     Area      Height     Area
  #   [min]        [min]   [mAU*s]     [mAU]        %
----|-------|----|-------|----------|----------|--------|
   1  10.599 BB    0.2575   31.70750    1.87327 100.0000

Totals :                    31.70750    1.87327


=====================================================================
                          *** End of Report ***
```

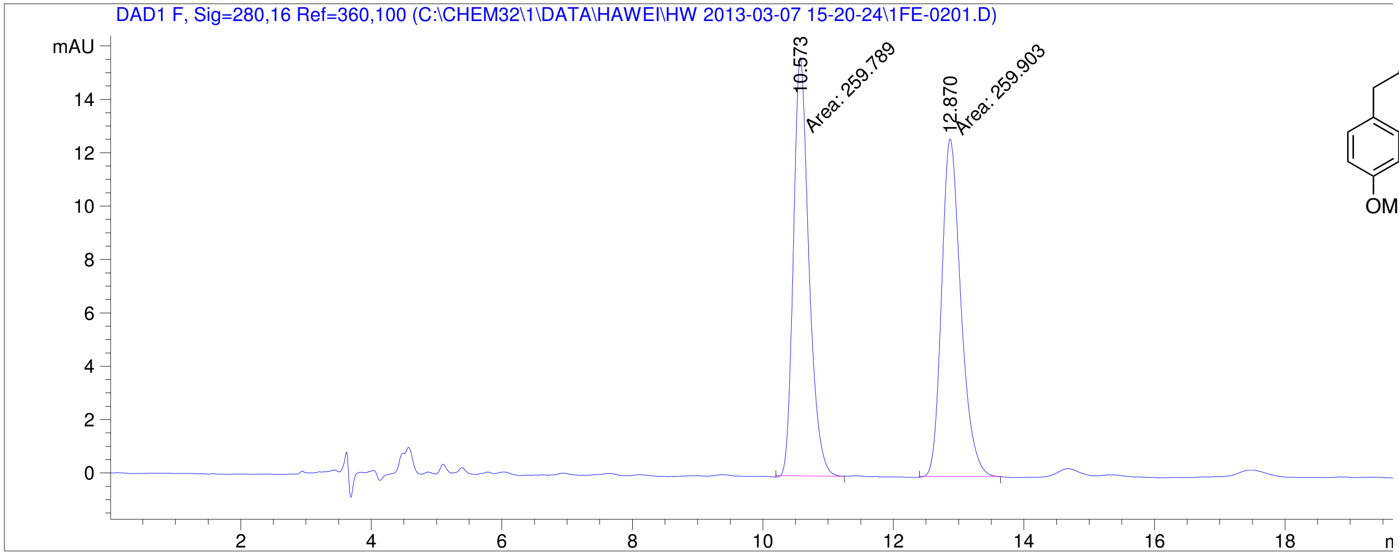

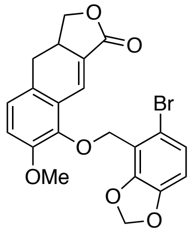

(±)-**2**

```
=====================================================================
                         Area Percent Report
=====================================================================

Sorted By             :      Signal
Multiplier            :      1.0000
Dilution              :      1.0000
Use Multiplier & Dilution Factor with ISTDs


Signal 1: DAD1 F, Sig=280,16 Ref=360,100

Peak RetTime Type  Width     Area      Height     Area
  #   [min]        [min]   [mAU*s]     [mAU]        %
----|-------|----|-------|----------|----------|--------|
   1  10.573 MM    0.2762  259.78934   15.67758  49.9890
   2  12.870 MM    0.3425  259.90326   12.64650  50.0110

Totals :                   519.69260   28.32407


=====================================================================
                          *** End of Report ***
```

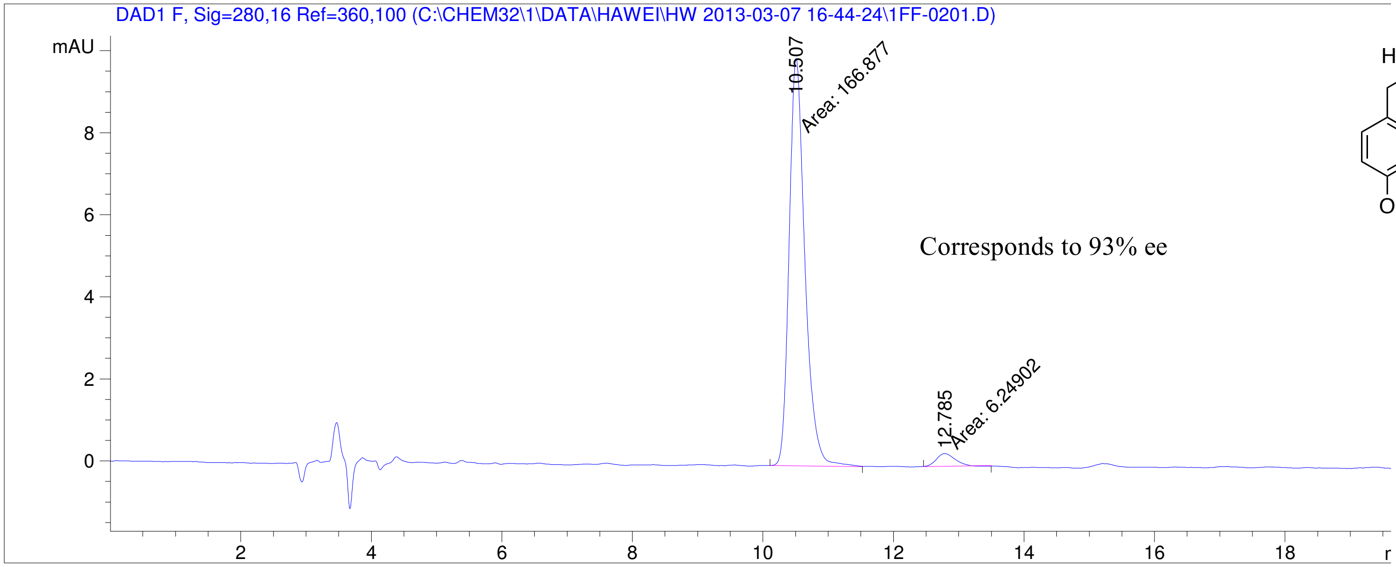

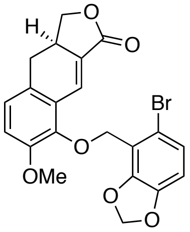

(+)-**2**

==

Area Percent Report

==

Sorted By : Signal
Multiplier : 1.0000
Dilution : 1.0000
Use Multiplier & Dilution Factor with ISTDs

Signal 1: DAD1 F, Sig=280,16 Ref=360,100

Peak #	RetTime [min]	Type	Width [min]	Area [mAU*s]	Height [mAU]	Area %
1	10.507	MM	0.2799	166.87708	9.93757	96.3905
2	12.785	MM	0.3325	6.24902	3.13201e-1	3.6095

Totals : 173.12610 10.25078

==

*** End of Report ***

NMR - Spectra

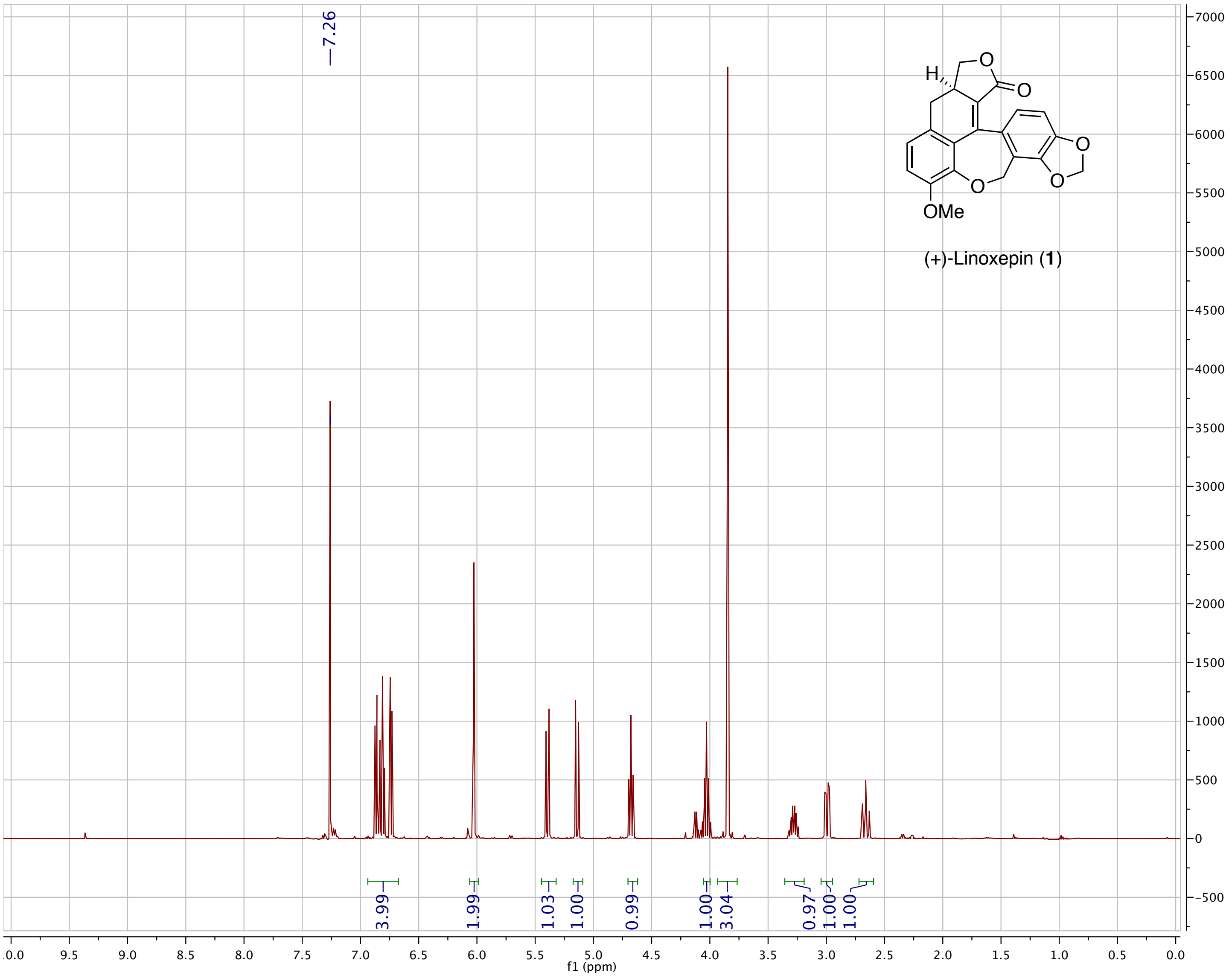

7.26
H
O
O
O
O
O
OMe
(+)-Linoxepin (1)
3.99
1.99
1.03
1.00
0.99
1.00
3.04
0.97
1.00
1.00
f1 (ppm)

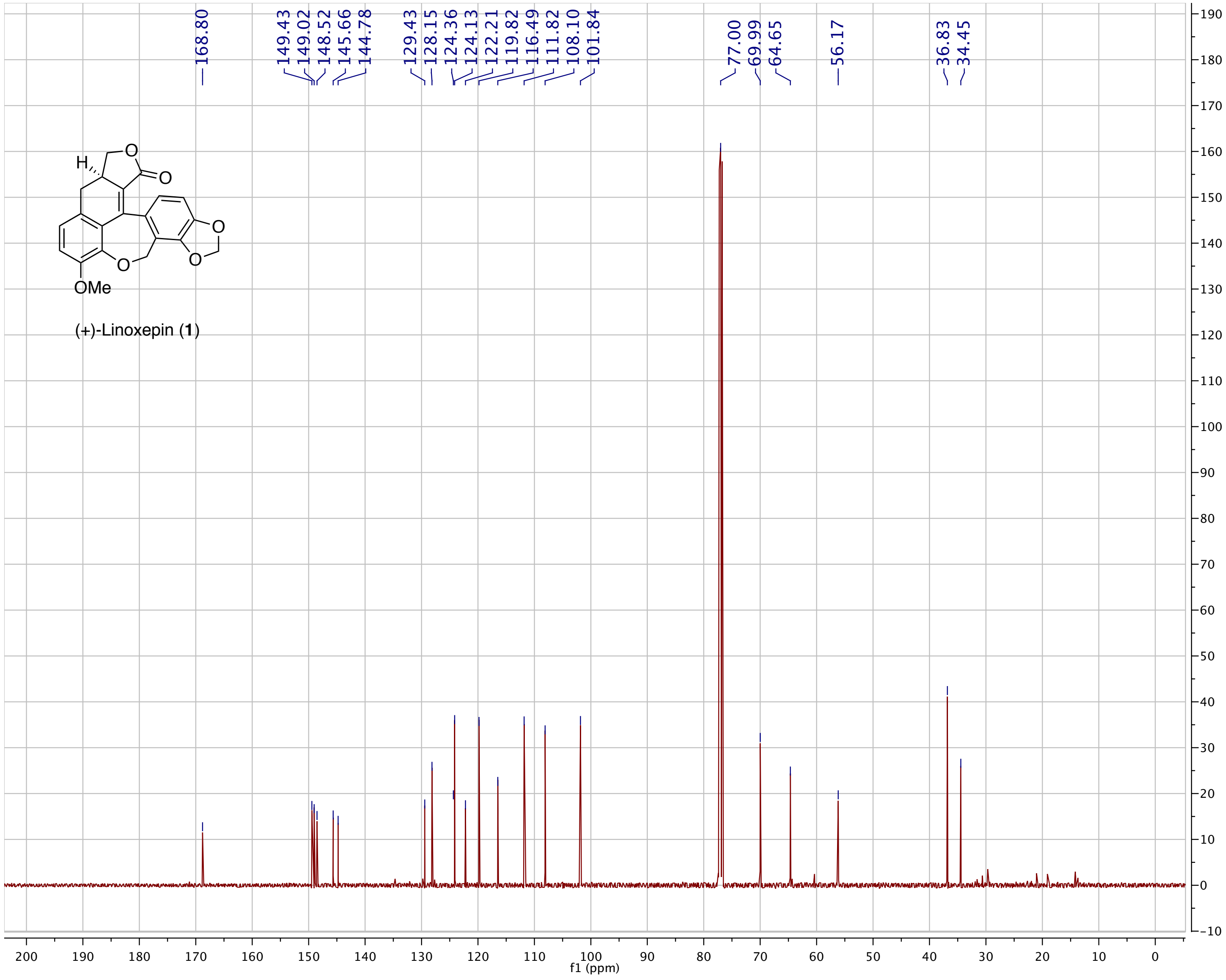
168.80
149.43
149.02
148.52
145.66
144.78
129.43
128.15
124.36
124.13
122.21
119.82
116.49
111.82
108.10
101.84
77.00
69.99
64.65
56.17
36.83
34.45
H
O
O
O
O
O
OMe
(+)-Linoxepin (1)
f1 (ppm)

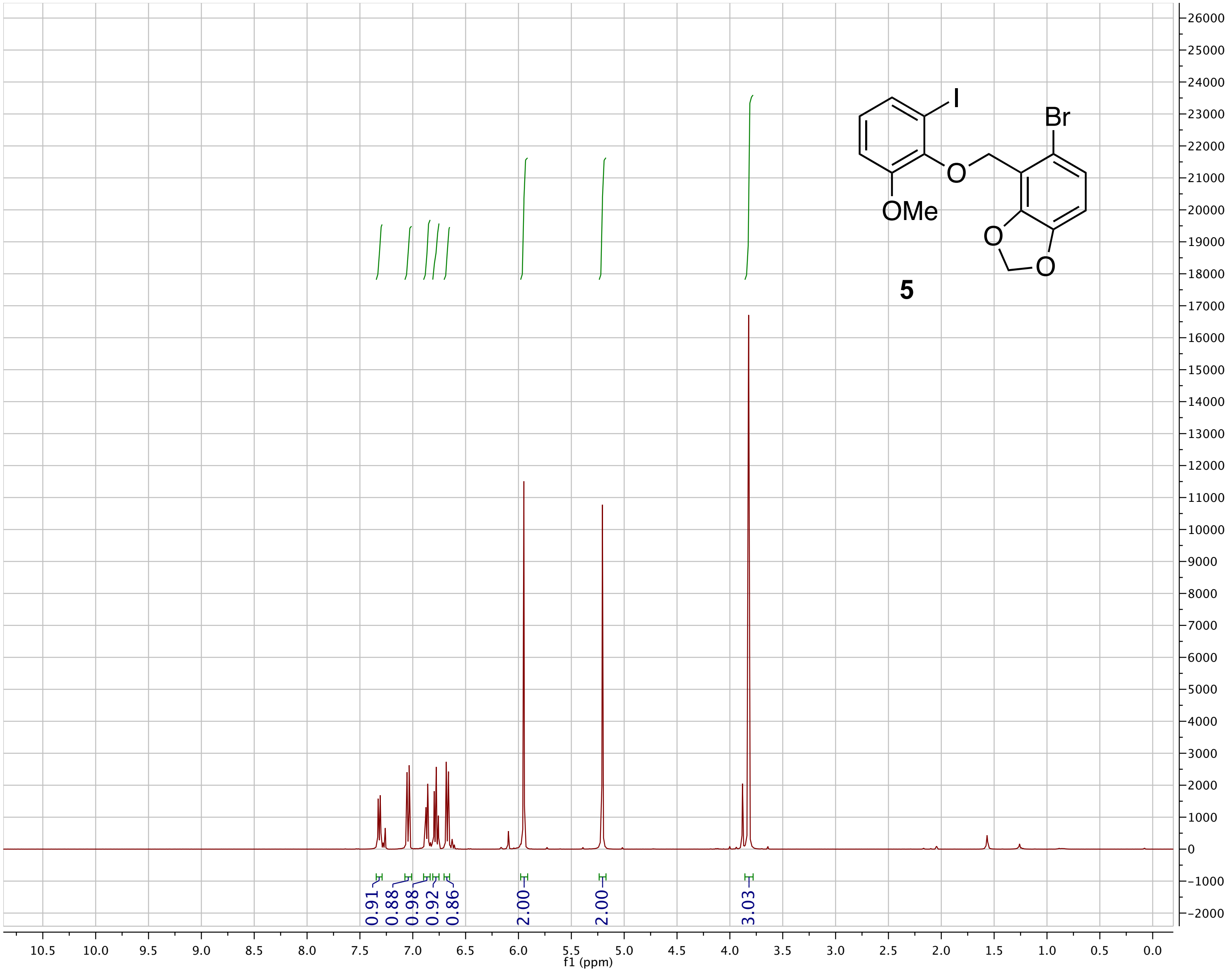
I
Br
O
OMe
O
O
5
f1 (ppm)
0.91
0.88
0.98
0.92
0.86
2.00
2.00
3.03

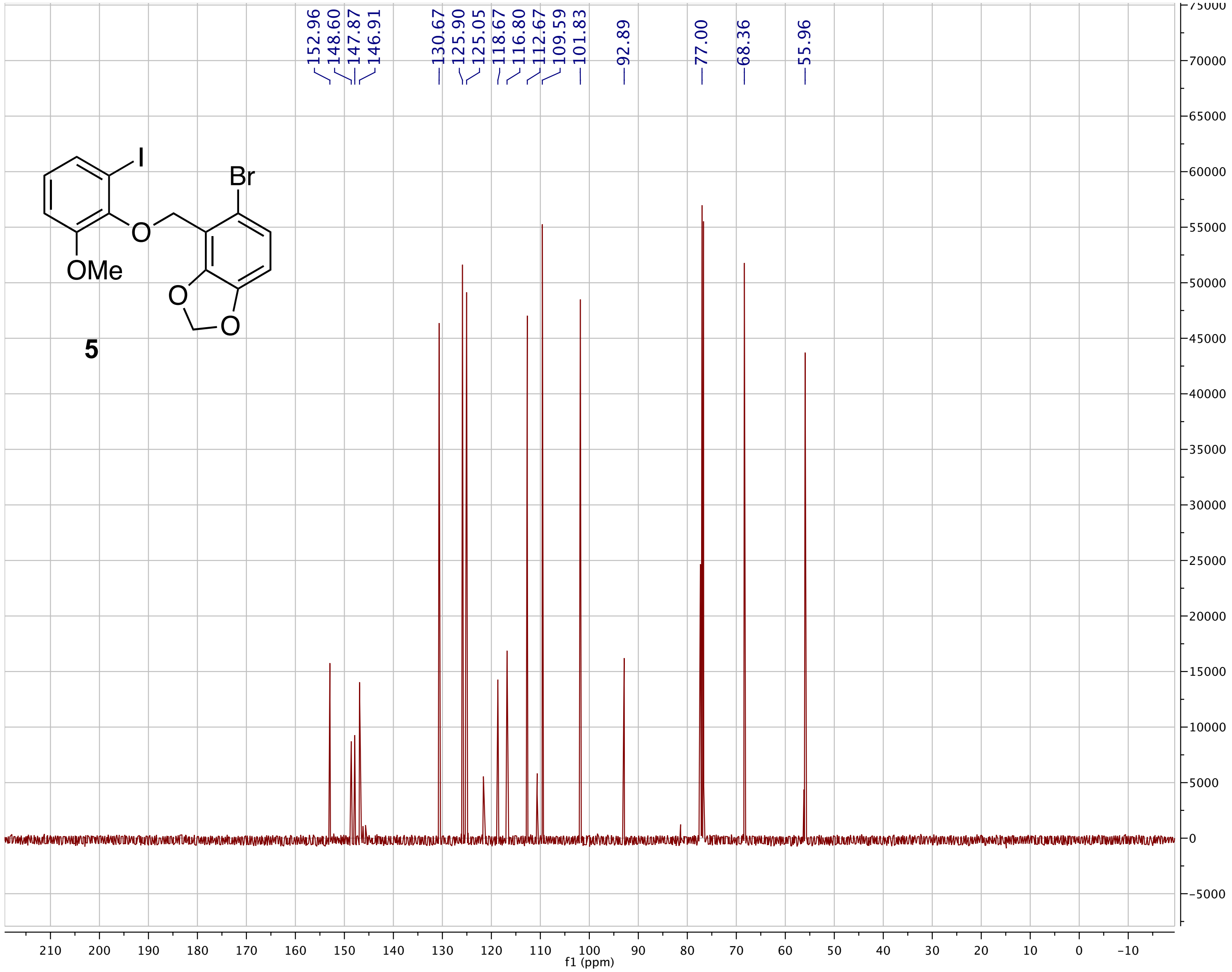
152.96
148.60
147.87
146.91
130.67
125.90
125.05
118.67
116.80
112.67
109.59
101.83
92.89
77.00
68.36
55.96
I
Br
O
OMe
O
O
5
f1 (ppm)

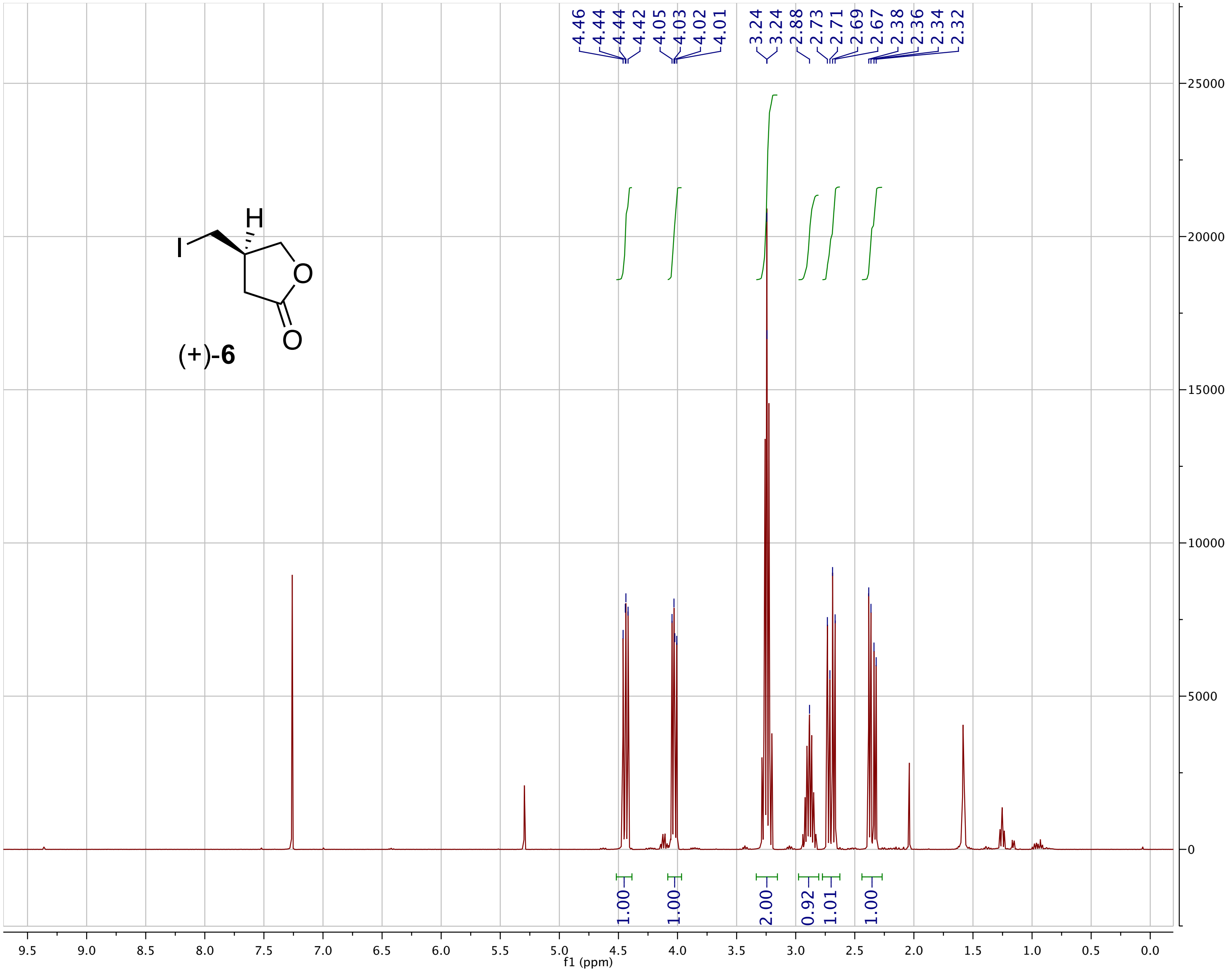

(+)-**6**

4.46
4.44
4.44
4.42
4.05
4.03
4.02
4.01
3.24
3.24
2.88
2.73
2.71
2.69
2.67
2.38
2.36
2.34
2.32
1.00
1.00
2.00
0.92
1.01
1.00
f1 (ppm)

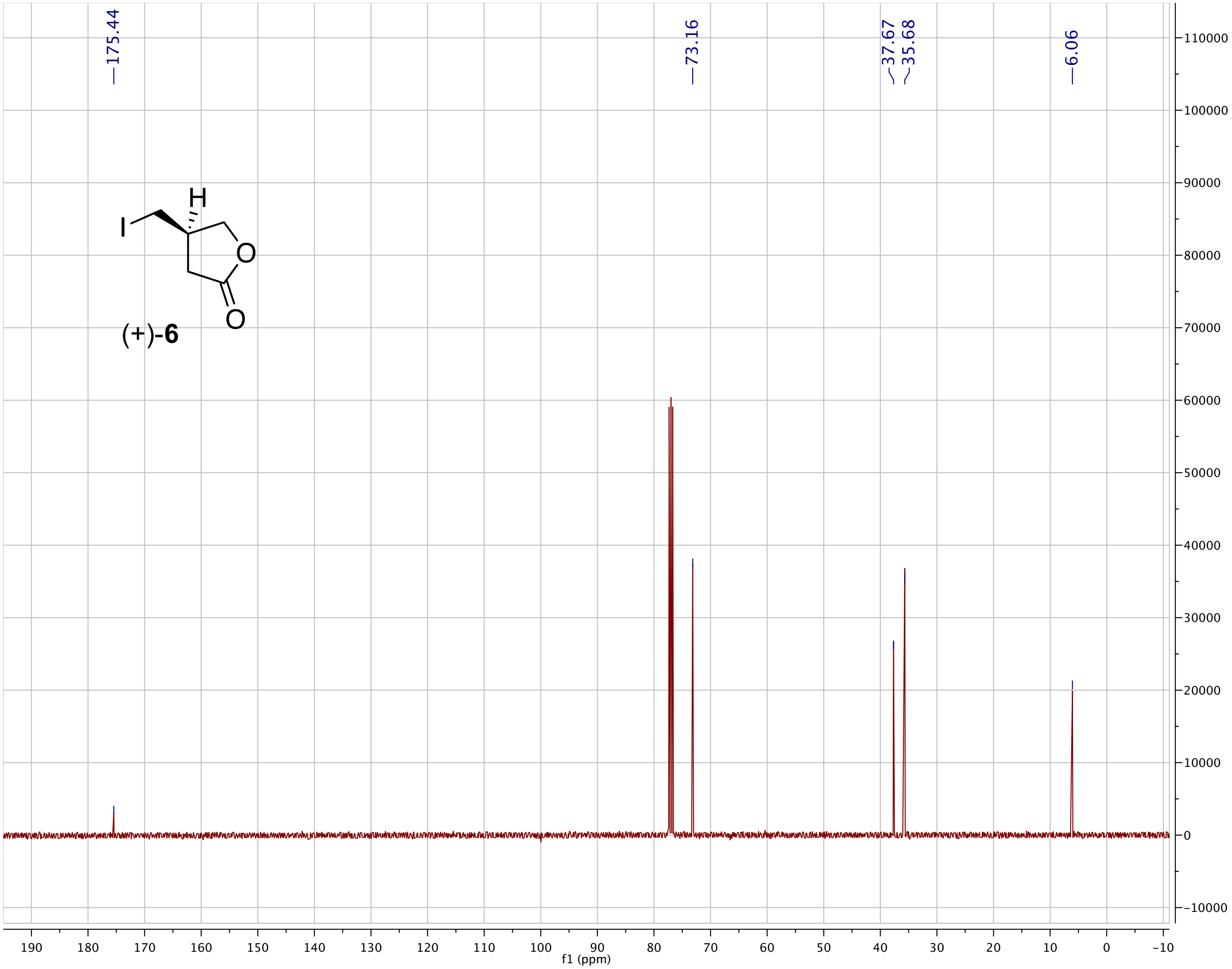

175.44
73.16
37.67
35.68
6.06
H
I
O
O
(+)-6
f1 (ppm)

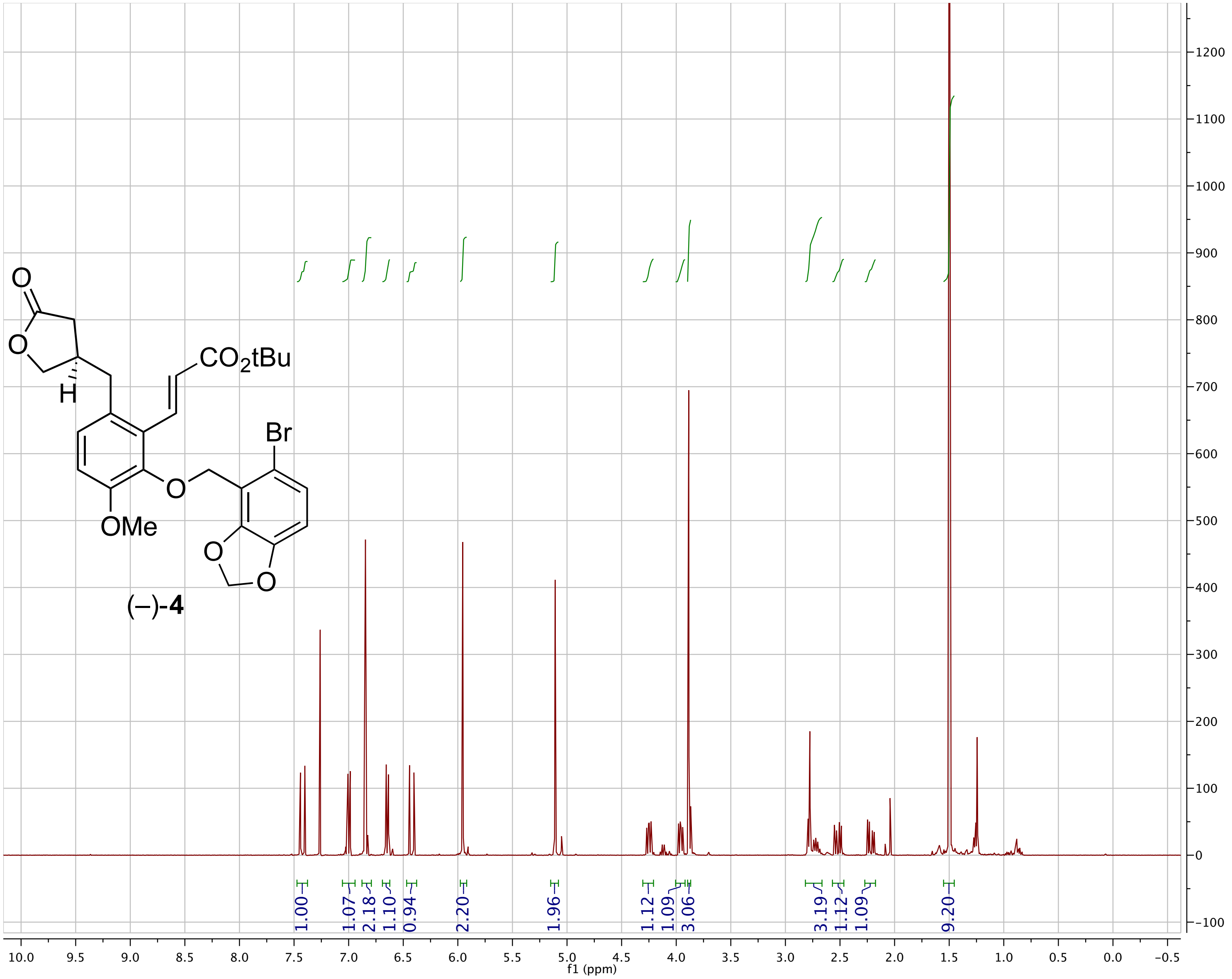

CO2tBu
Br
OMe
(–)-4
1.00
1.07
2.18
1.10
0.94
2.20
1.96
1.12
1.09
3.06
3.19
1.12
1.09
9.20
f1 (ppm)

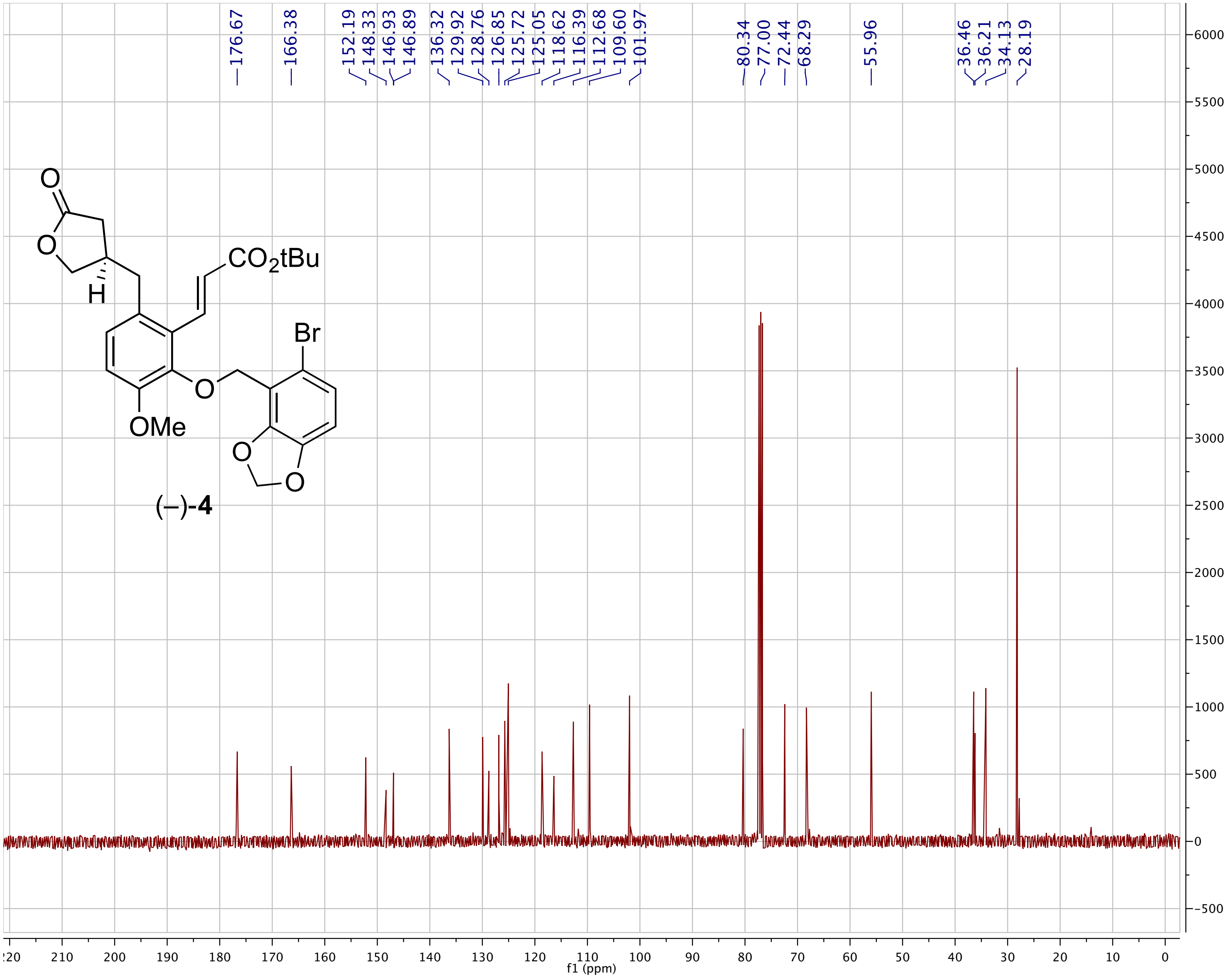
176.67
166.38
152.19
148.33
146.93
146.89
136.32
129.92
128.76
126.85
125.72
125.05
118.62
116.39
112.68
109.60
101.97
80.34
77.00
72.44
68.29
55.96
36.46
36.21
34.13
28.19
CO_2tBu
Br
OMe
(–)-4
f1 (ppm)

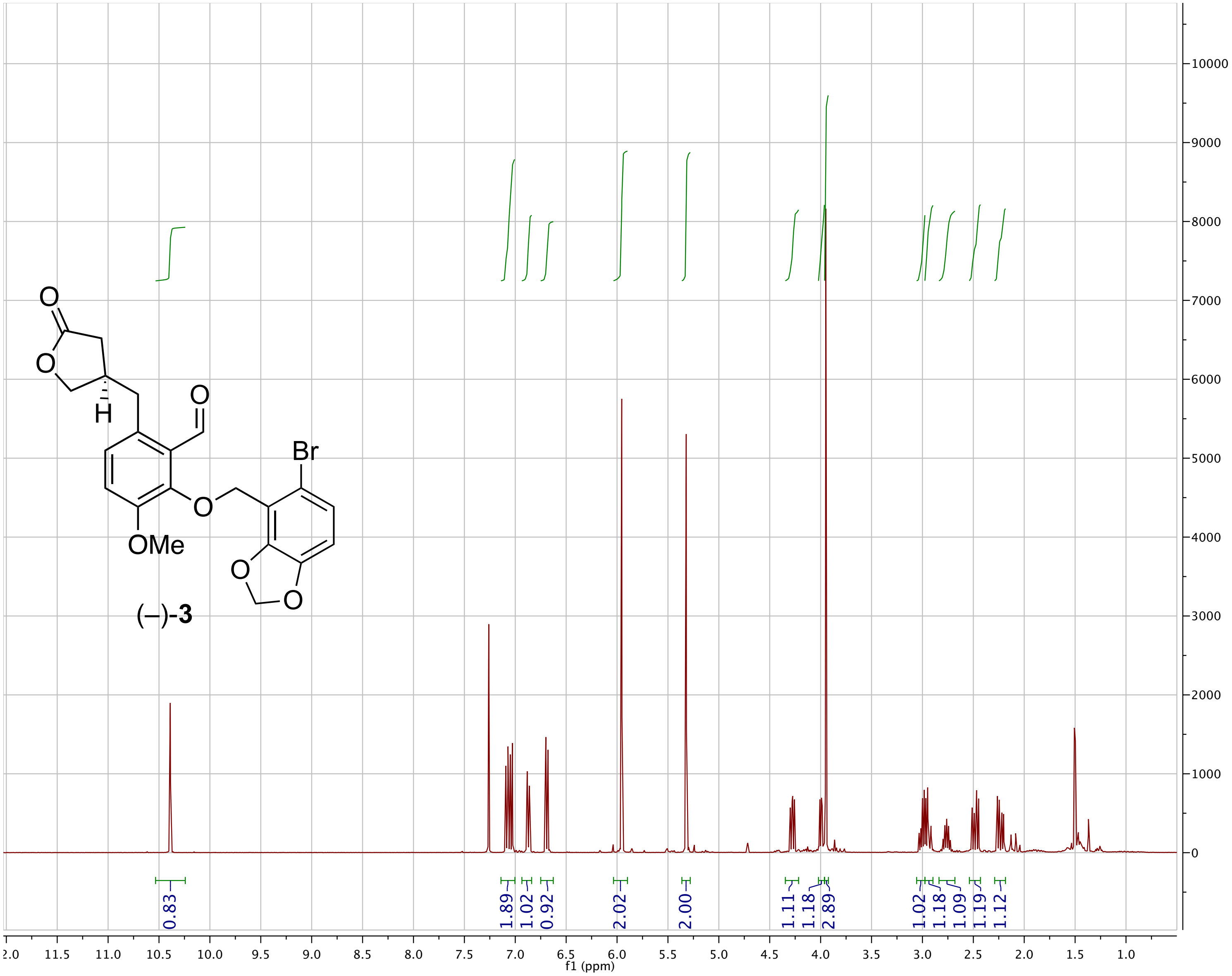
(–)-3
0.83
1.89
1.02
0.92
2.02
2.00
1.11
1.18
2.89
1.02
1.18
1.09
1.19
1.12
f1 (ppm)

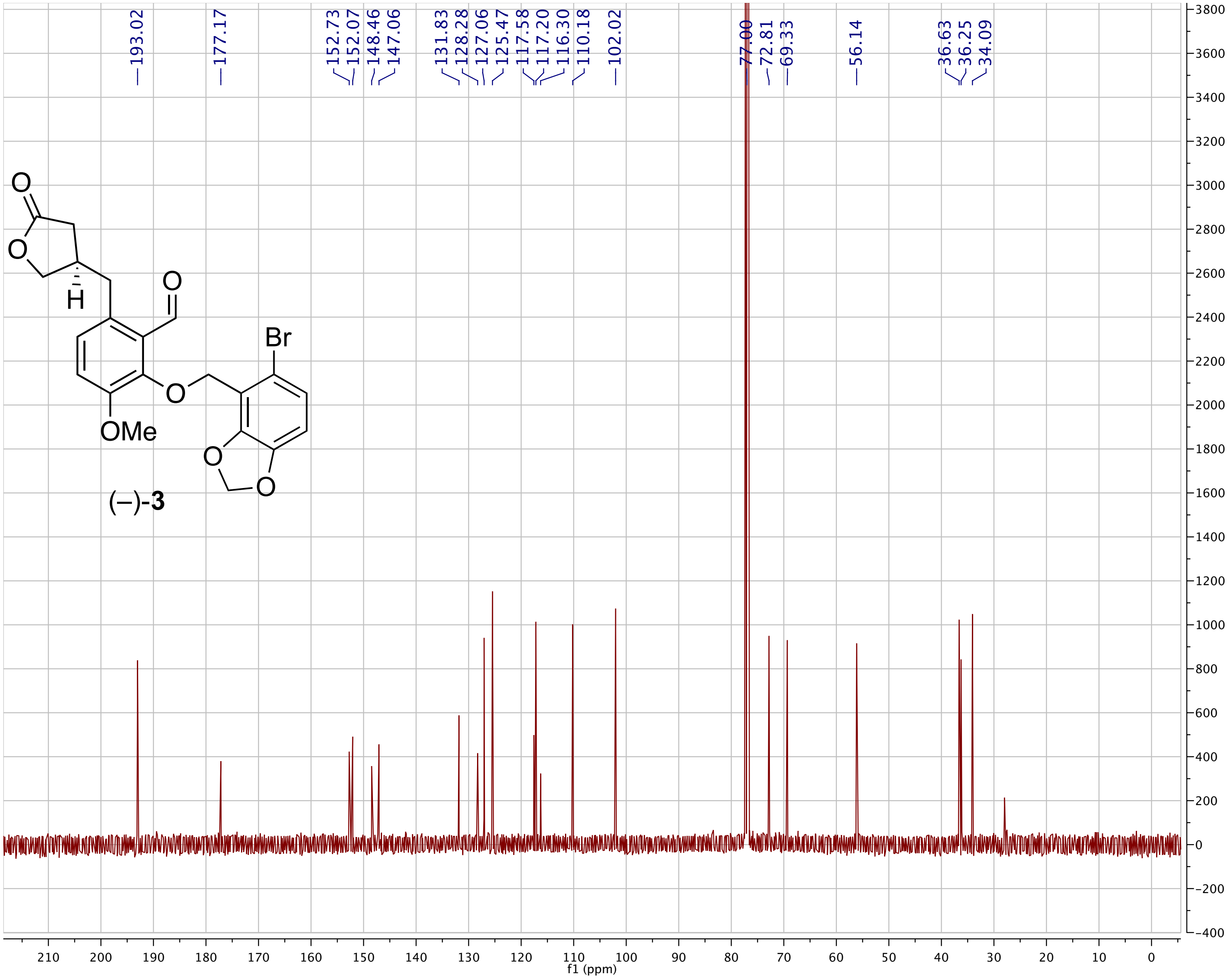

193.02
177.17
152.73
152.07
148.46
147.06
131.83
128.28
127.06
125.47
117.58
117.20
116.30
110.18
102.02
77.00
72.81
69.33
56.14
36.63
36.25
34.09
Br
OMe
(−)-3
f1 (ppm)

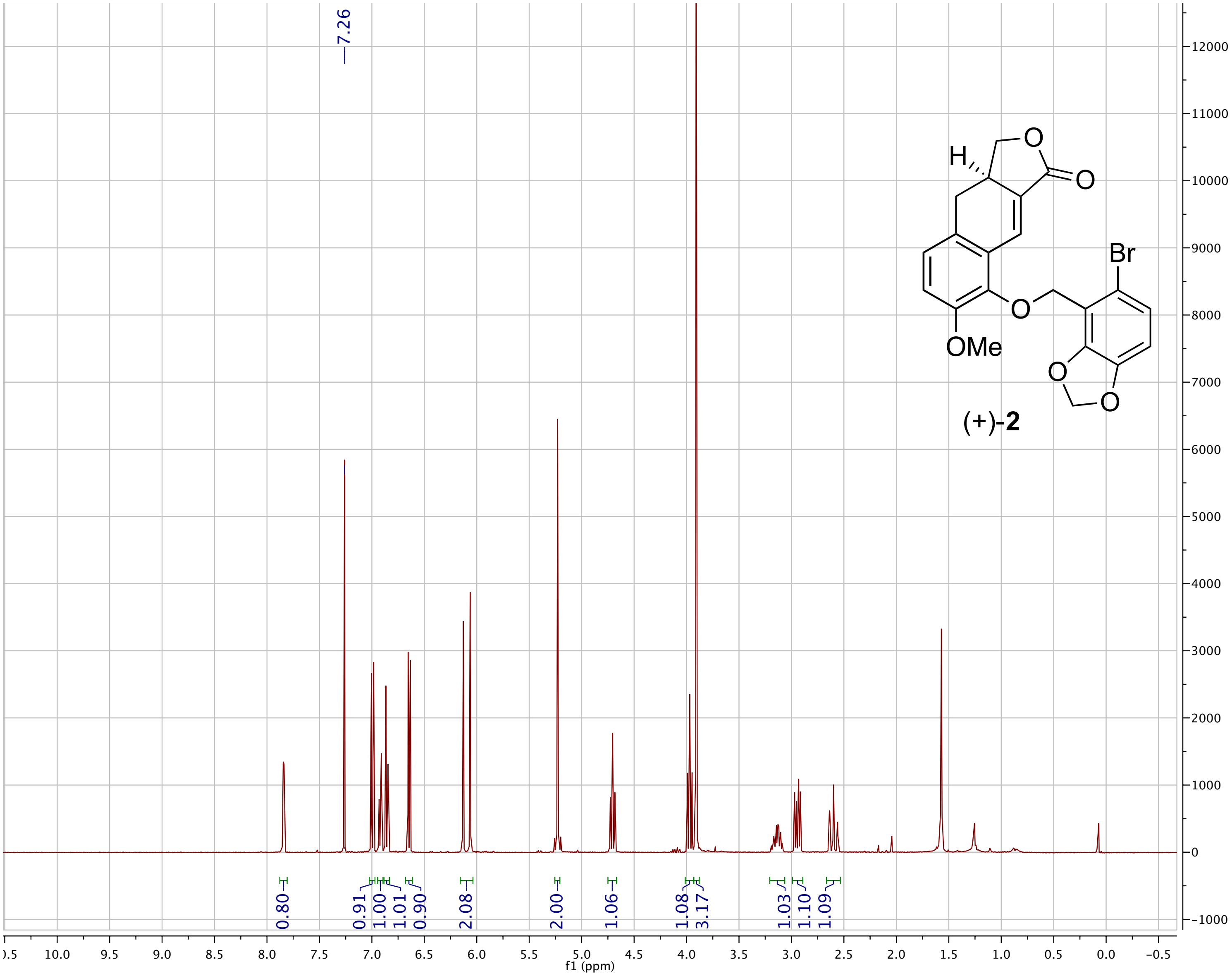

7.26
0.80
0.91
1.00
1.01
0.90
2.08
2.00
1.06
1.08
3.17
1.03
1.10
1.09
f1 (ppm)
H
O
O
Br
O
OMe
O
O
(+)-2

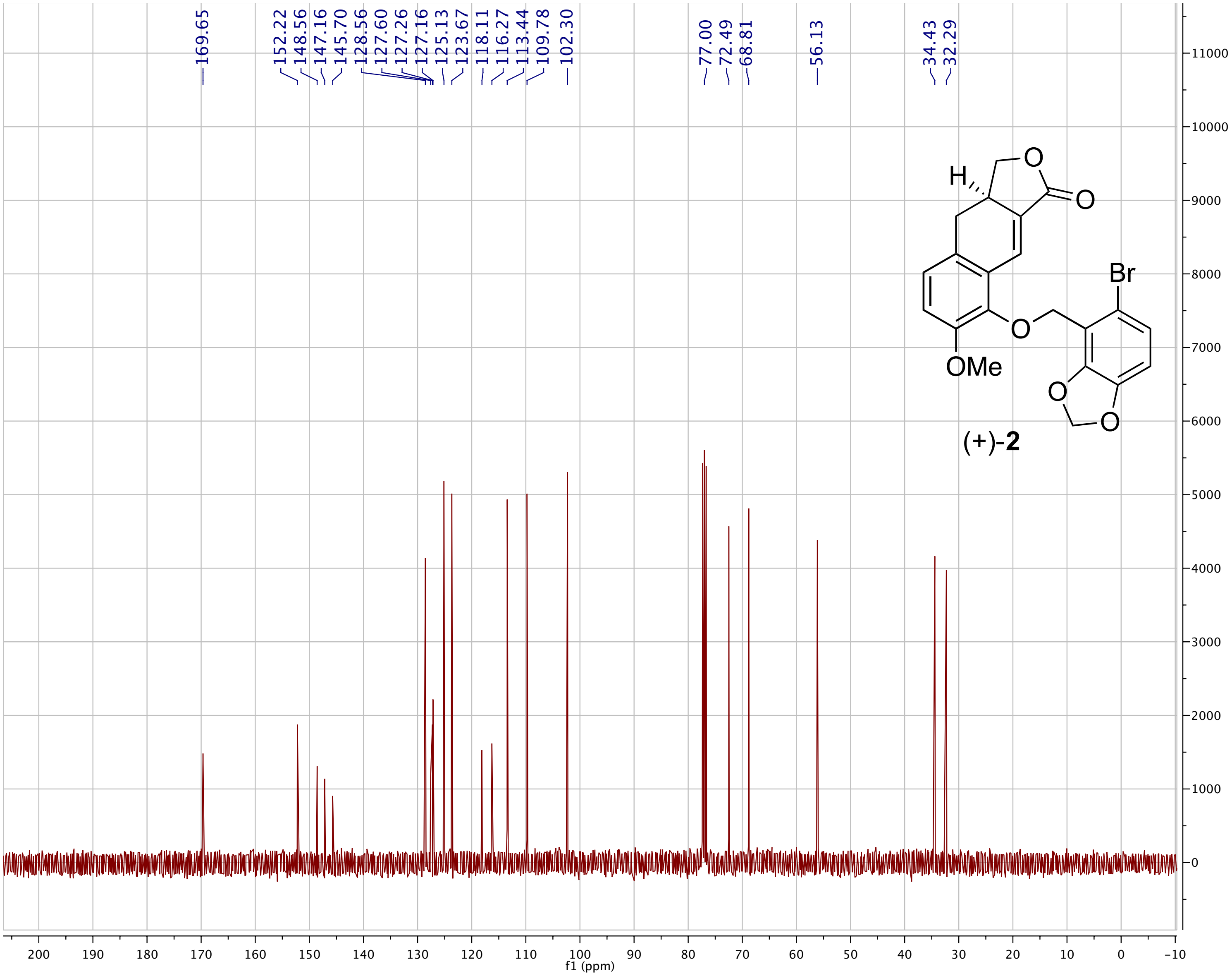
169.65
152.22
148.56
147.16
145.70
128.56
127.60
127.26
127.16
125.13
123.67
118.11
116.27
113.44
109.78
102.30
77.00
72.49
68.81
56.13
34.43
32.29
H
O
O
Br
O
OMe
O
O
(+)-2
f1 (ppm)

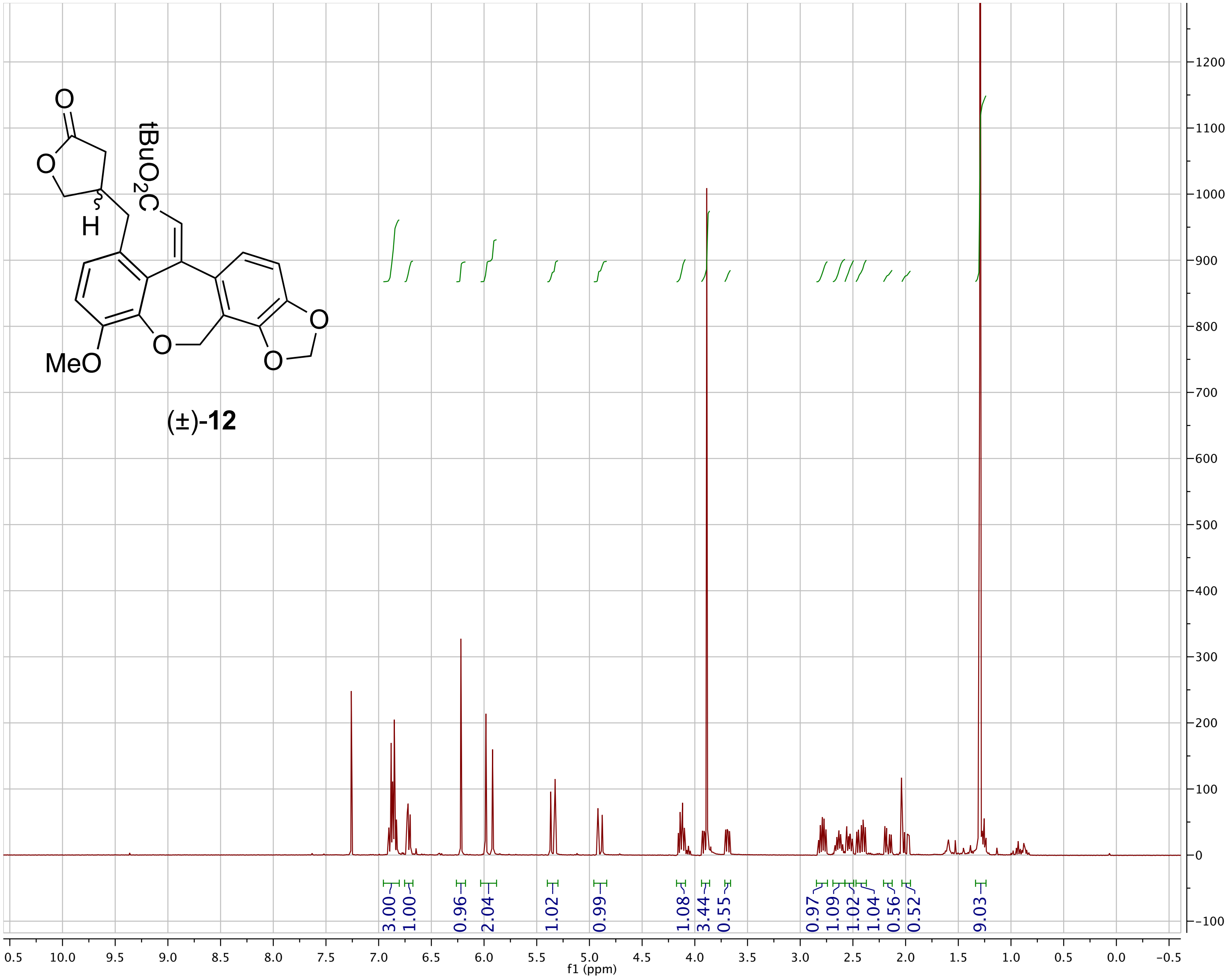

The occurrence of rotamers cause a duplication of signals

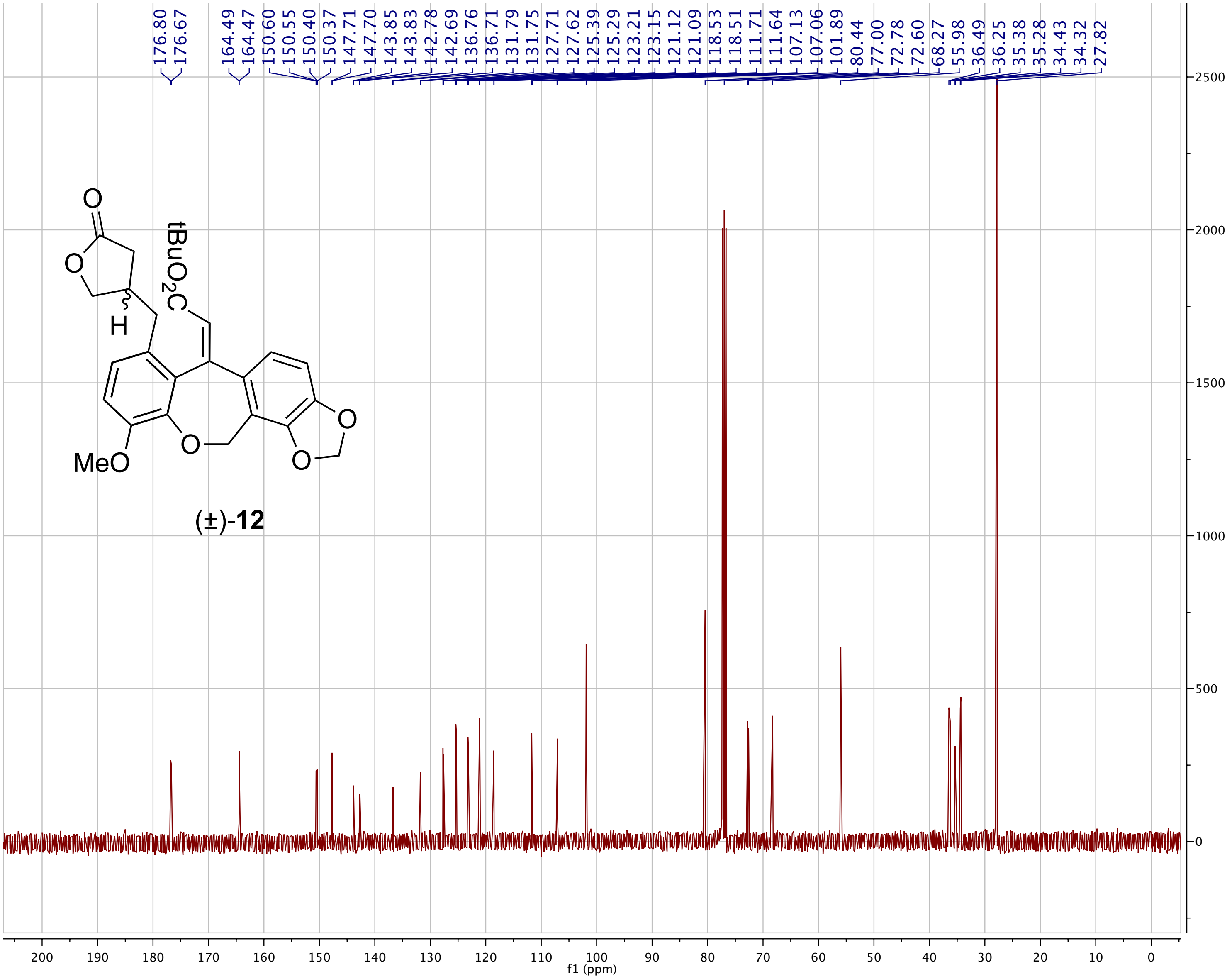

The occurrence of rotamers cause a duplication of signals

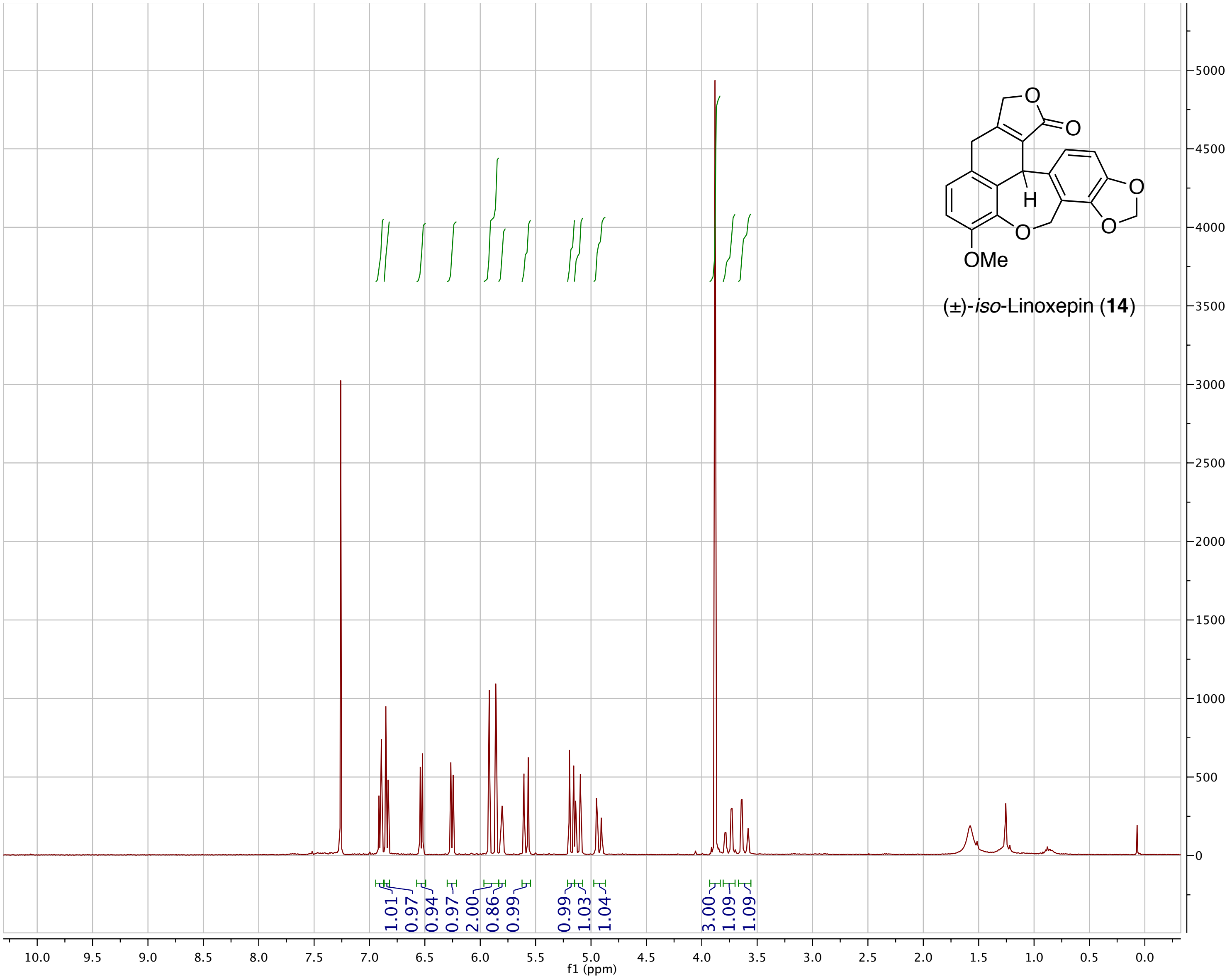
(±)-*iso*-Linoxepin (**14**)
OMe
H
1.01
0.97
0.94
0.97
2.00
0.86
0.99
0.99
1.03
1.04
3.00
1.09
1.09
f1 (ppm)

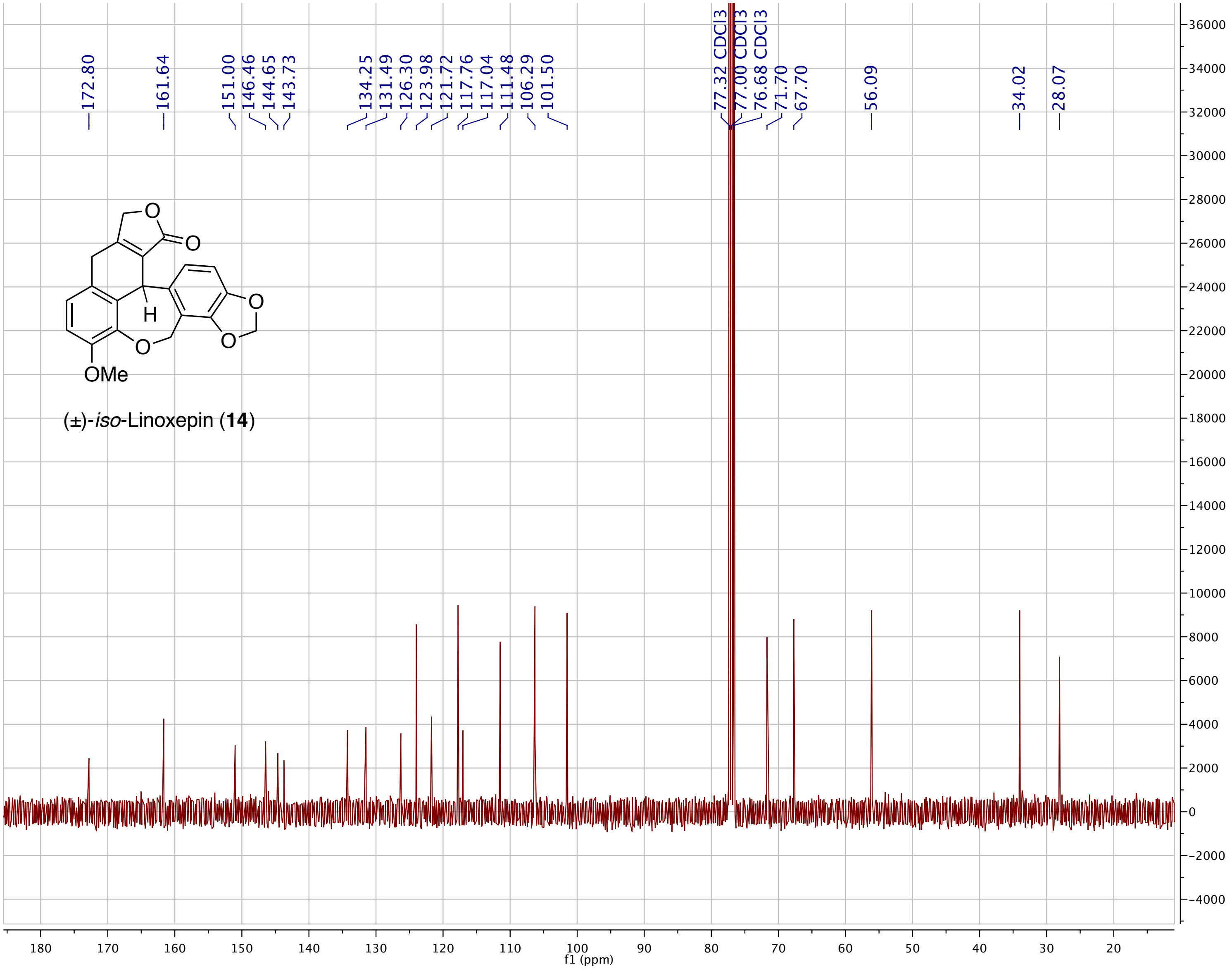
172.80
161.64
151.00
146.46
144.65
143.73
134.25
131.49
126.30
123.98
121.72
117.76
117.04
111.48
106.29
101.50
77.32 CDCl3
77.00 CDCl3
76.68 CDCl3
71.70
67.70
56.09
34.02
28.07
OMe
(±)-*iso*-Linoxepin (**14**)
f1 (ppm)
180
170
160
150
140
130
120
110
100
90
80
70
60
50
40
30
20
36000
34000
32000
30000
28000
26000
24000
22000
20000
18000
16000
14000
12000
10000
8000
6000
4000
2000
0
−2000
−4000

X-ray crystal structures

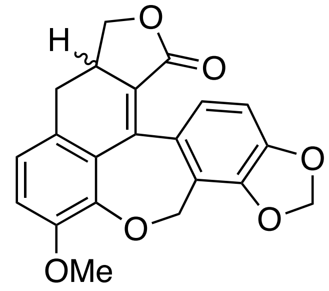

(±)-linoxepin (**1**)

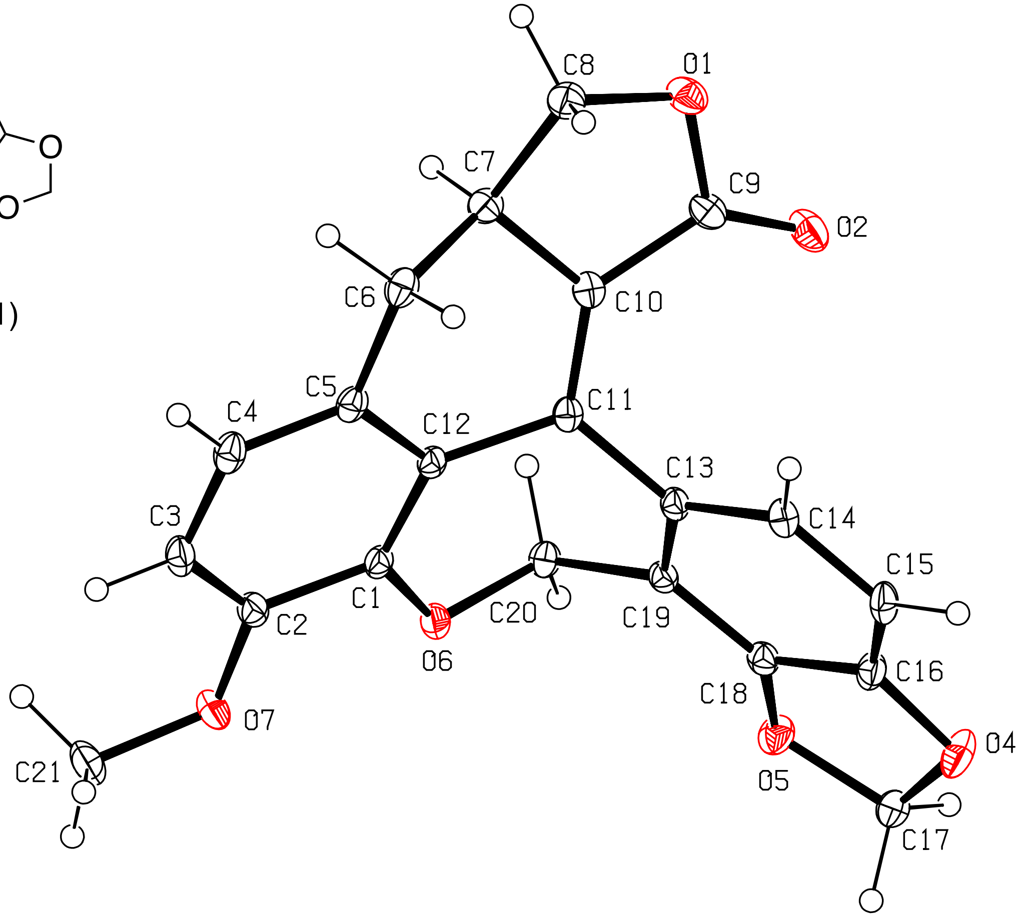

Identification code	d12229 (Linoxepin, **1**)	
Empirical formula	C21 H16 O6	
Formula weight	364.34	
Temperature	147(2) K	
Wavelength	0.71073 Å	
Crystal system	Monoclinic	
Space group	P 21/n	
Unit cell dimensions	a = 11.598(3) Å	a= 90°.
	b = 8.3156(18) Å	b= 107.341(4)°.
	c = 17.301(4) Å	g = 90°.
Volume	1592.7(6) $Å^3$	
Z	4	
Density (calculated)	1.519 Mg/m^3	
Absorption coefficient	0.112 mm^{-1}	
F(000)	760	
Crystal size	0.22 x 0.21 x 0.15 mm^3	
Theta range for data collection	1.88 to 27.55°.	
Index ranges	-15<=h<=15, -10<=k<=10, -22<=l<=22	
Reflections collected	25823	
Independent reflections	3663 [R(int) = 0.0352]	
Completeness to theta = 27.55°	99.5 %	
Absorption correction	Semi-empirical from equivalents	
Max. and min. transmission	0.7456 and 0.7095	
Refinement method	Full-matrix least-squares on F^2	
Data / restraints / parameters	3663 / 0 / 245	
Goodness-of-fit on F^2	1.048	
Final R indices [I>2sigma(I)]	R1 = 0.0373, wR2 = 0.0971	
R indices (all data)	R1 = 0.0459, wR2 = 0.1028	
Largest diff. peak and hole	0.332 and -0.214 e.$Å^{-3}$	

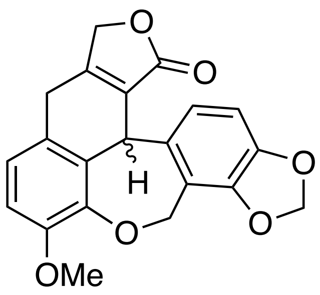

(±)-*iso*-linoxepin (**14**)

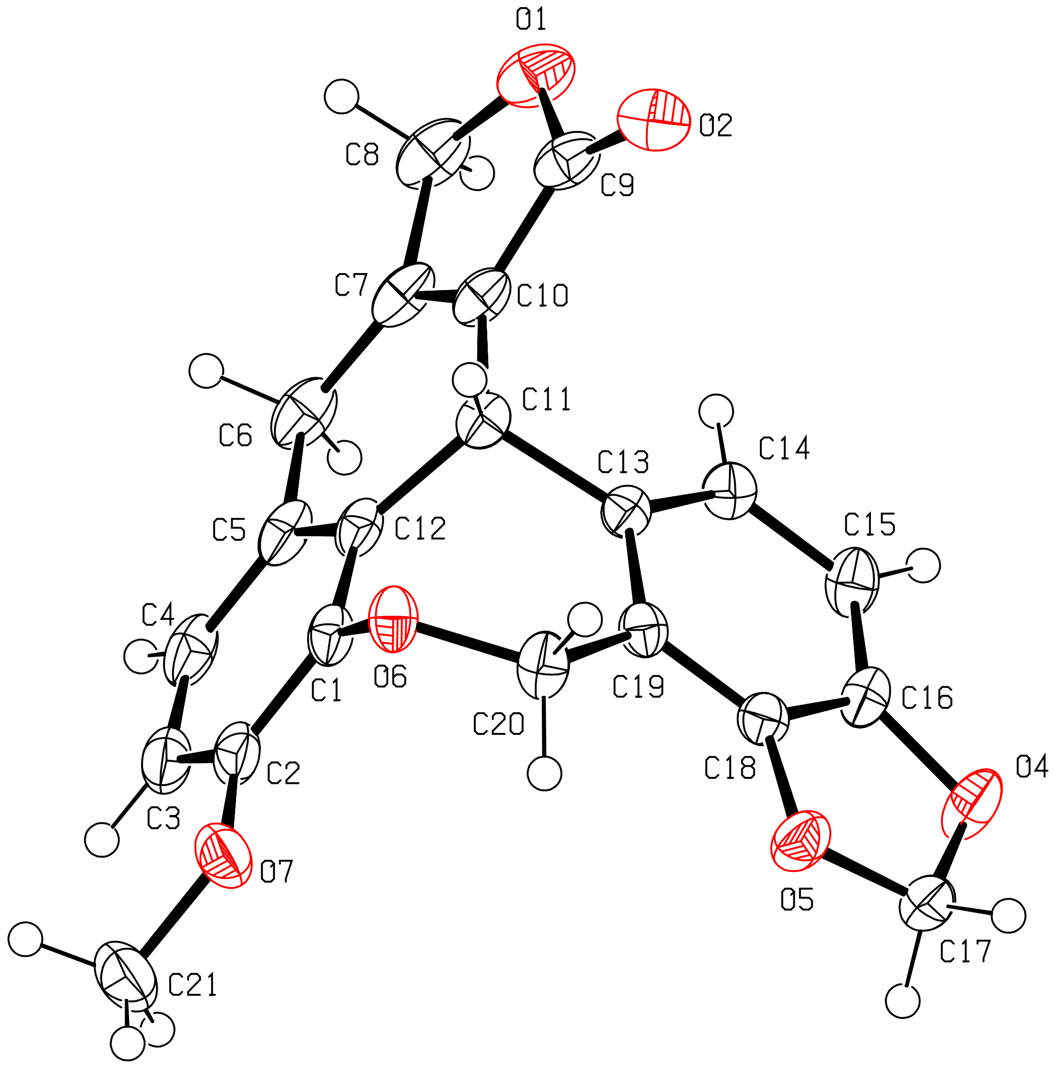

Identification code	d12260 (Isolinoxepin, **14**)	
Empirical formula	C21 H16 O6	
Formula weight	364.34	
Temperature	147(2) K	
Wavelength	1.54178 Å	
Crystal system	Orthorhombic	
Space group	P b c a	
Unit cell dimensions	a = 7.3087(1) Å	a= 90°.
	b = 20.7381(4) Å	b= 90°.
	c = 20.9623(4) Å	g = 90°.
Volume	3177.23(10) $Å^3$	
Z	8	
Density (calculated)	1.523 Mg/m^3	
Absorption coefficient	0.937 mm^{-1}	
F(000)	1520	
Crystal size	0.21 x 0.08 x 0.07 mm^3	
Theta range for data collection	4.22 to 66.49°.	
Index ranges	-6<=h<=8, -22<=k<=24, -19<=l<=24	
Reflections collected	10742	
Independent reflections	2675 [R(int) = 0.0324]	
Completeness to theta = 66.49°	95.5 %	
Absorption correction	Semi-empirical from equivalents	
Max. and min. transmission	0.7528 and 0.6827	
Refinement method	Full-matrix least-squares on F^2	
Data / restraints / parameters	2675 / 0 / 245	
Goodness-of-fit on F^2	1.046	
Final R indices [I>2sigma(I)]	R1 = 0.0352, wR2 = 0.0876	
R indices (all data)	R1 = 0.0390, wR2 = 0.0909	
Largest diff. peak and hole	0.172 and -0.196 e.$Å^{-3}$	

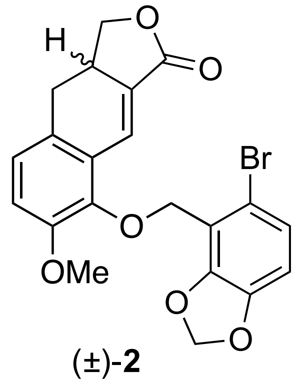
H
O
O
Br
O
OMe
O
O

(±)-**2**

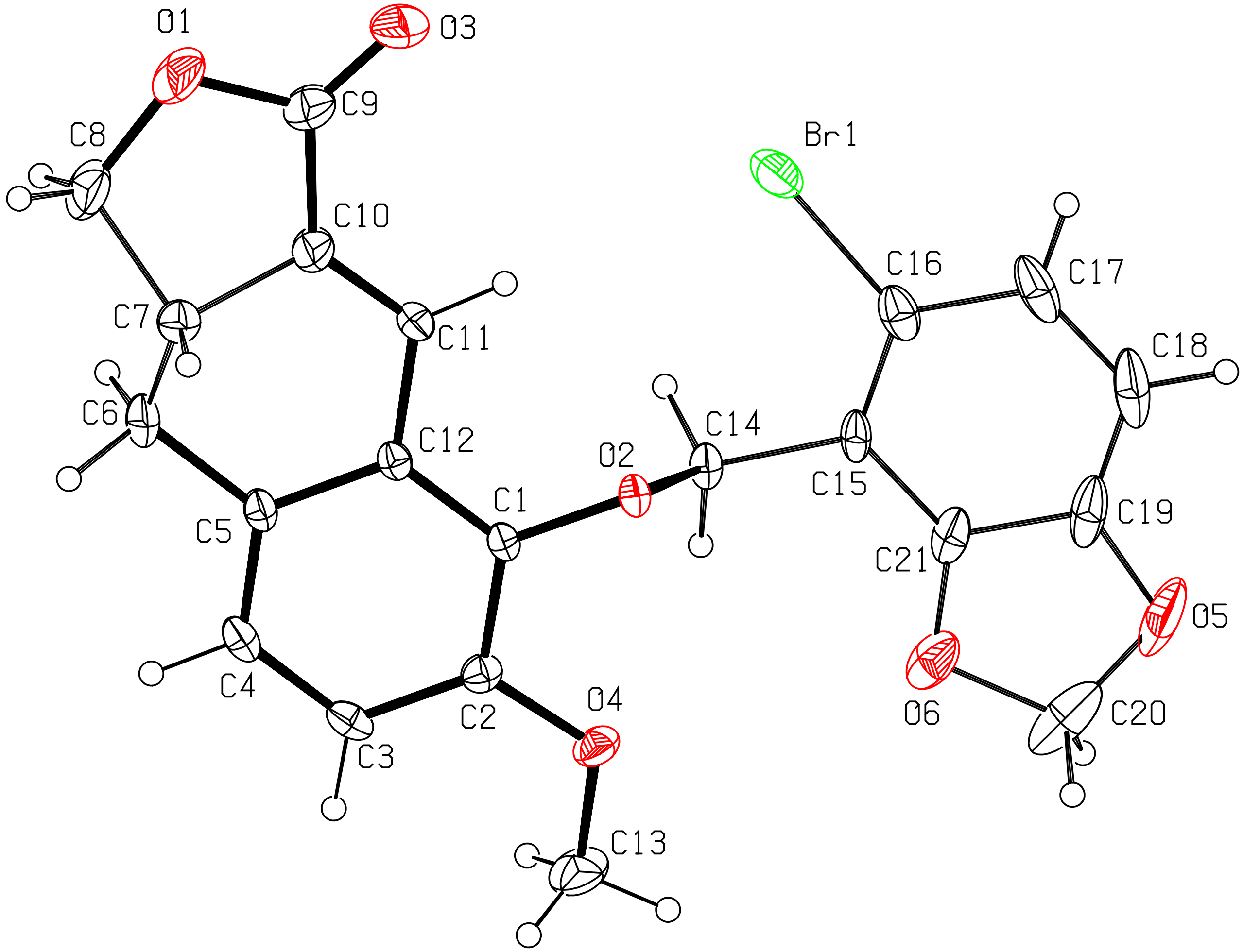
O1
O3
C9
C8
C10
C7
C11
C6
C12
C5
C1
O2
C14
C15
Br1
C16
C17
C18
C19
C21
O5
O6
C20
C4
C3
C2
O4
C13

Identification code	d12200 (compound **2**)	
Empirical formula	C21 H17 Br O6	
Formula weight	445.26	
Temperature	147(2) K	
Wavelength	1.54178 Å	
Crystal system	Orthorhombic	
Space group	P b c a	
Unit cell dimensions	a = 19.694(1) Å	a= 90°.
	b = 7.3470(4) Å	b= 90°.
	c = 25.0384(13) Å	g = 90°.
Volume	3622.9(3) $Å^3$	
Z	8	
Density (calculated)	1.633 Mg/m^3	
Absorption coefficient	3.428 mm^{-1}	
F(000)	1808	
Crystal size	0.23 x 0.11 x 0.03 mm^3	
Theta range for data collection	3.53 to 66.72°.	
Index ranges	-23<=h<=23, -8<=k<=8, -29<=l<=29	
Reflections collected	21720	
Independent reflections	3163 [R(int) = 0.0380]	
Completeness to theta = 66.72°	98.6 %	
Absorption correction	Semi-empirical from equivalents	
Max. and min. transmission	0.7528 and 0.6169	
Refinement method	Full-matrix least-squares on F^2	
Data / restraints / parameters	3163 / 38 / 274	
Goodness-of-fit on F^2	1.115	
Final R indices [I>2sigma(I)]	R1 = 0.0510, wR2 = 0.1179	
R indices (all data)	R1 = 0.0535, wR2 = 0.1193	
Largest diff. peak and hole	0.805 and -0.500 e.$Å^{-3}$	

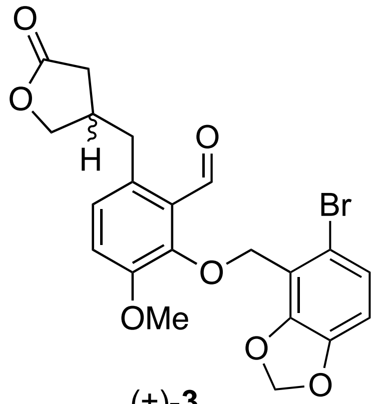
O
O
H
O
Br
O
OMe
O
O

(±)-**3**

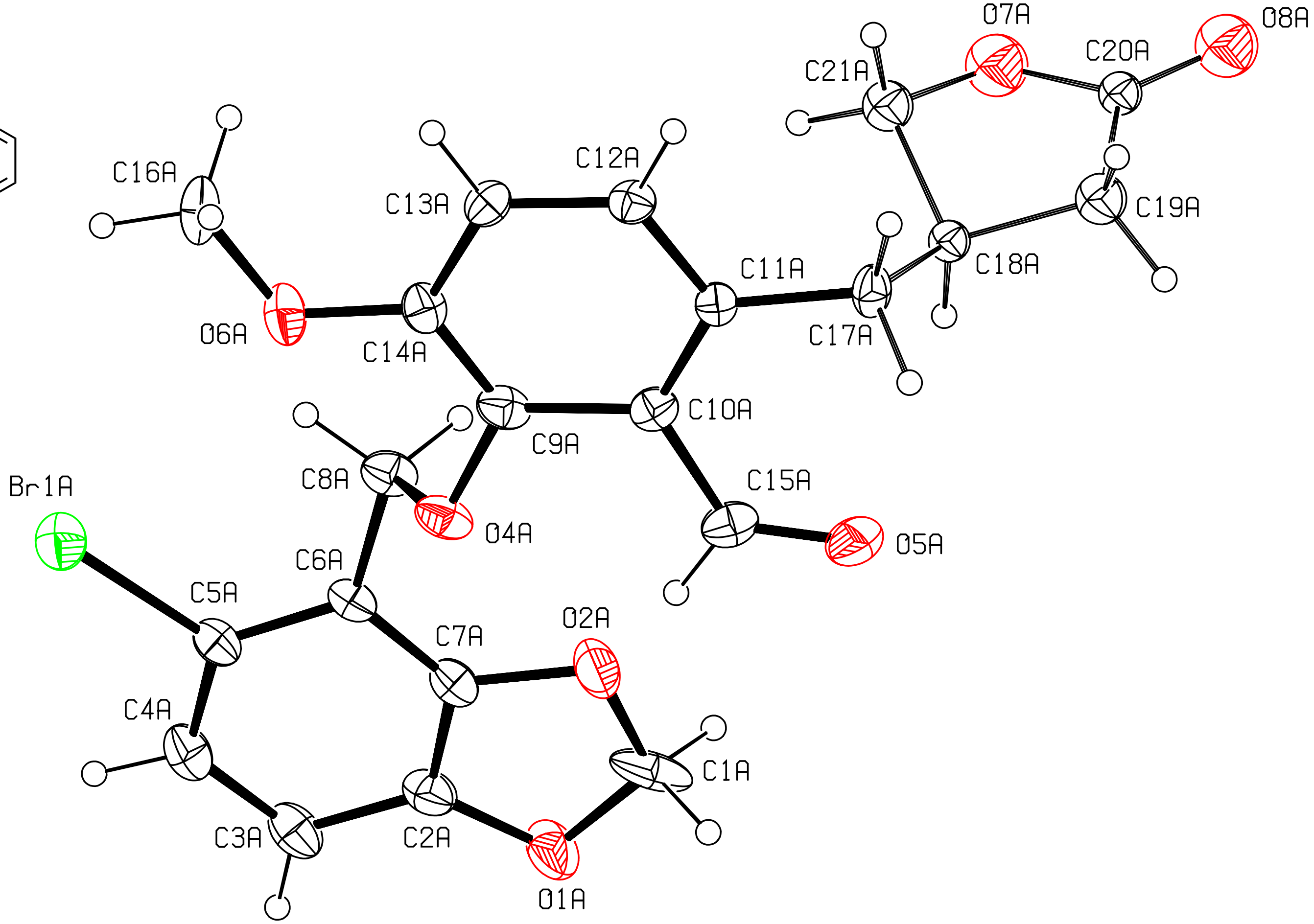
C16A
C13A
C12A
C21A
O7A
C20A
O8A
C19A
C18A
C11A
C17A
O6A
C14A
C9A
C10A
C8A
Br1A
O4A
C15A
O5A
C6A
C5A
C7A
O2A
C4A
C1A
C3A
C2A
O1A

Identification code	d12144 (compound **3**)	
Empirical formula	C21 H19 Br O7	
Formula weight	463.27	
Temperature	147(2) K	
Wavelength	1.54178 Å	
Crystal system	Orthorhombic	
Space group	P c a 21	
Unit cell dimensions	a = 13.5951(4) Å	a= 90°.
	b = 5.3632(2) Å	b= 90°.
	c = 51.7142(17) Å	g = 90°.
Volume	3770.6(2) Å^3	
Z	8	
Density (calculated)	1.632 Mg/m^3	
Absorption coefficient	3.358 mm^{-1}	
F(000)	1888	
Crystal size	0.10 x 0.07 x 0.07 mm^3	
Theta range for data collection	3.42 to 66.16°.	
Index ranges	-15<=h<=16, -6<=k<=4, -61<=l<=56	
Reflections collected	13743	
Independent reflections	5625 [R(int) = 0.0261]	
Completeness to theta = 66.16°	97.0 %	
Absorption correction	Semi-empirical from equivalents	
Max. and min. transmission	0.7527 and 0.6662	
Refinement method	Full-matrix least-squares on F^2	
Data / restraints / parameters	5625 / 33 / 513	
Goodness-of-fit on F^2	1.023	
Final R indices [I>2sigma(I)]	R1 = 0.0485, wR2 = 0.1314	
R indices (all data)	R1 = 0.0504, wR2 = 0.1336	
Absolute structure parameter	0.51(2)	
Largest diff. peak and hole	0.779 and -0.590 e.Å^{-3}	

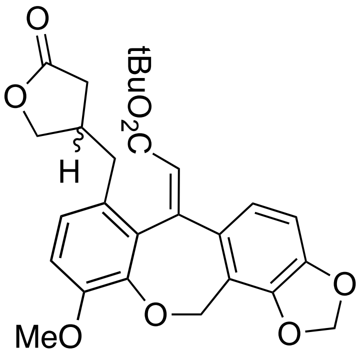

O
O
tBuO2C
H
MeO
O
O
O

(±)-12

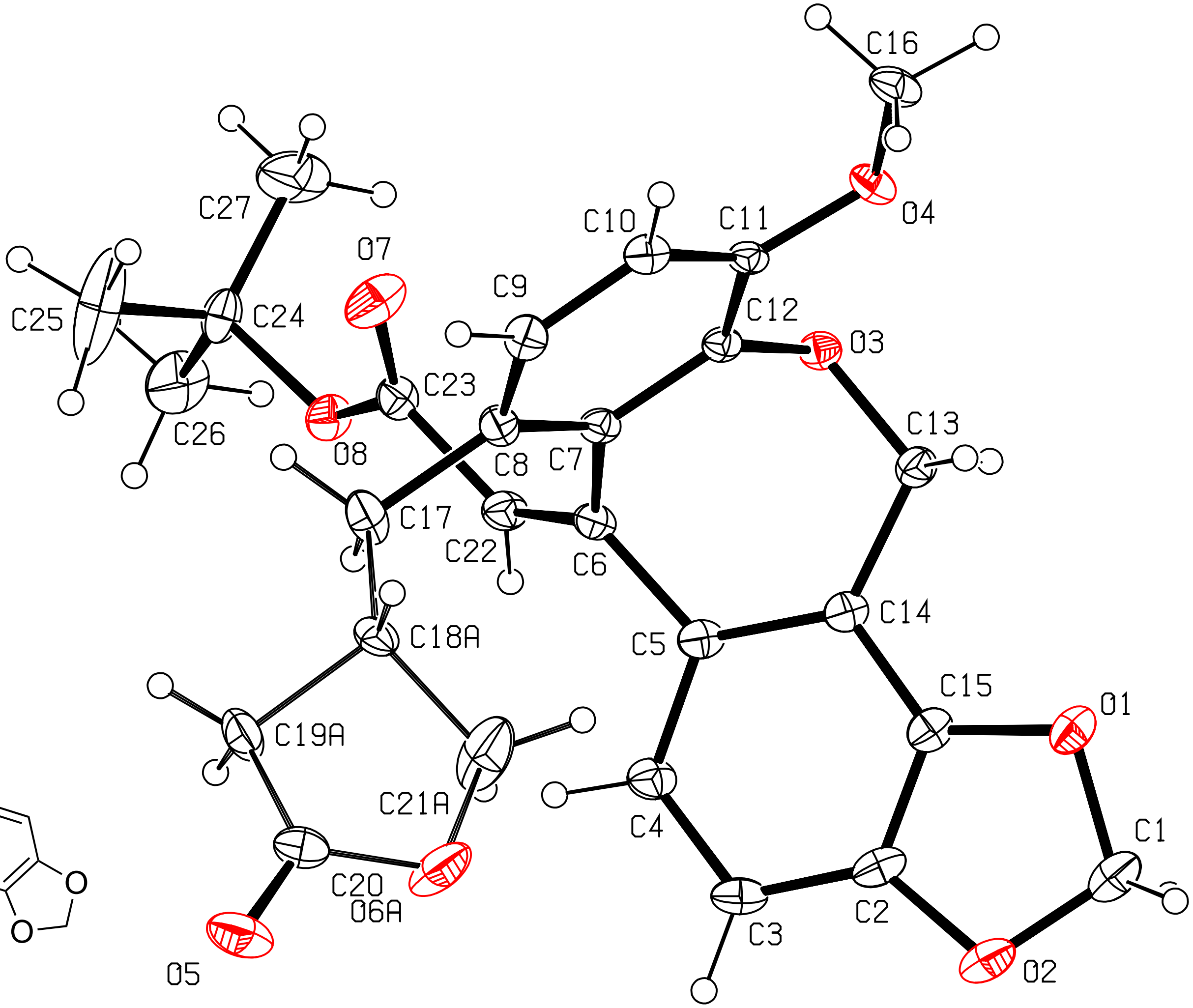

C16
O4
C11
C10
C27
O7
C9
C12
O3
C25
C24
C23
C26
O8
C8
C7
C13
C17
C22
C6
C14
C18A
C5
C15
O1
C19A
C21A
C4
C1
C20
O6A
C3
C2
O2
O5

Identification code	d12143 (compound **12**)	
Empirical formula	C27 H28 O8	
Formula weight	480.49	
Temperature	147(2) K	
Wavelength	0.71073 Å	
Crystal system	Monoclinic	
Space group	P 21/c	
Unit cell dimensions	a = 10.7906(9) Å	a= 90°.
	b = 13.0218(11) Å	b= 97.152(2)°.
	c = 16.9171(14) Å	g = 90°.
Volume	2358.6(3) Å^3	
Z	4	
Density (calculated)	1.353 Mg/m^3	
Absorption coefficient	0.100 mm^{-1}	
F(000)	1016	
Crystal size	0.17 x 0.15 x 0.11 mm^3	
Theta range for data collection	1.90 to 27.54°.	
Index ranges	-14<=h<=14, -16<=k<=16, -20<=l<=21	
Reflections collected	22095	
Independent reflections	5407 [R(int) = 0.0405]	
Completeness to theta = 27.54°	99.7 %	
Absorption correction	Semi-empirical from equivalents	
Max. and min. transmission	0.7456 and 0.7094	
Refinement method	Full-matrix least-squares on F^2	
Data / restraints / parameters	5407 / 6 / 345	
Goodness-of-fit on F^2	1.006	
Final R indices [I>2sigma(I)]	R1 = 0.0475, wR2 = 0.1135	
R indices (all data)	R1 = 0.0724, wR2 = 0.1266	
Largest diff. peak and hole	0.375 and -0.437 e.Å^{-3}	
